# In silico identification of compounds from *Piper sarmentosum* Roxb leaf fractionated extract inhibit interleukin-6 to prevent rheumatoid arthritis

**DOI:** 10.3389/fphar.2024.1358037

**Published:** 2024-03-21

**Authors:** Tran Nhat Phong Dao, Sunday Amos Onikanni, Adewale Oluwaseun Fadaka, Ashwil Klein, Van De Tran, Minh Hoang Le, Chih-Hao Wang, Hen-Hong Chang

**Affiliations:** ^1^ Graduate Institute of Integrated Medicine, College of Chinese Medicine, China Medical University, Taichung, Taiwan; ^2^ Faculty of Traditional Medicine, Can Tho University of Medicine and Pharmacy, Can Tho, Vietnam; ^3^ College of Medicine, Graduate Institute of Biomedical Sciences, China Medical University, Taichung, Taiwan; ^4^ Department of Chemical Sciences, Biochemistry Unit, Afe-Babalola University, Ado-Ekiti, Nigeria; ^5^ Department of Biotechnology, University of the Western Cape, Bellville, South Africa; ^6^ Department of Health Organization and Management, Can Tho University of Medicine and Pharmacy, Can Tho, Vietnam; ^7^ Graduate Institute of Cell Biology, China Medical University, Taichung, Taiwan; ^8^ Chinese Medicine Research Center, China Medical University, Taichung, Taiwan; ^9^ Department of Chinese Medicine, China Medical University Hospital, Taichung, Taiwan

**Keywords:** rheumatoid arthritis, interleukin-6, phytonutrient, simulation, conformational

## Abstract

**Objective:** Medicinal herbs with a phytonutrient background has been applied globally as major alternatives to ameliorate the continuous increase in rheumatoid arthritis cases worldwide. We herein aimed to critically examine the bioactive components of the medicinal herb Piper sarmentosum Roxb leaf fractionated extract for its potential to inhibit the influx of interleukin-6 (IL-6) in rheumatoid arthritis.

**Methods:** The Schrödinger platform was employed as the main computational acumen for the screening of bioactive compounds identified and reference compounds subjected to molecular simulation (MDS) for analyzing the stability of docked complexes to assess fluctuations and conformational changes during protein–ligand interactions.

**Results:** The values of the simulatory properties and principal component analysis (PCA) revealed the good stability of these phytochemicals in the active pocket of interleukin-6 (IL-6).

**Discussion:** Our findings reveal new strategies in which these phytochemicals are potential inhibitory agents that can be modified and further evaluated to develop more effective agents for the management of rheumatoid arthritis, thereby providing a better understanding and useful model for the reproduction and/or discovery of new drugs for the management of rheumatoid arthritis and its complications.

## 1 Introduction

Rheumatoid arthritis (RA) is known as inflammatory arthropathy and is characterized by the presence of long-standing chronic autoimmune inflammatory disease primarily affecting synovial joints, leading to cartilage damage and bone erosion—a disabling and painful condition that can result in significant loss of function and mobility if left untreated (Scott et al., 2010; Amaya-Amaya et al., 2012). The protracted effect of this inflammatory disorder has been reported to ameliorate systemic challenges in several tissues and organs, thereby causing serious effects that eventually result in serious comorbidities such as cardiovascular diseases, osteoporosis, fractures, cognitive dysfunction, and depression (Amaya-Amaya et al., 2013). Research has shown the predominance and disease status of RA on account of geographic region to region, with higher estimates in industrialized countries and urban areas generally being greater. RA can manifest at any age, but its prevalence is greater among middle-aged individuals, particularly females (Finckh et al., 2022).

Furthermore, genetic factors have been identified as one of the major contributors to the pathogenic effect of RA, with higher heritability estimates for seropositive rheumatoid arthritis patients than for seronegative patients (McInnes and Schett, 2011; Aletaha and Smolen, 2018). However, given substantial recent advancements in understanding RA pathogenesis, this condition is incurable. Therefore, early evaluation, diagnosis, and management are paramount for achieving optimal therapeutic outcomes. Interleukin-6 (IL-6) is a pivotal immunomodulatory cytokine that plays a crucial role in the pathogenesis of various diseases, including autoimmune disorders, chronic inflammatory conditions, and cancer (Hirano, 2021). In the context of RA pathogenesis, IL-6 plays a central role. IL-6 interacts with various cell types, including B cells, T cells, osteoclasts, osteoblasts, osteocytes, and synovial fibroblasts, thereby triggering the migration of neutrophils into joints and the transition from acute to chronic inflammation (Srirangan and Choy, 2010; Takeuchi et al., 2021).

Moreover, in the B and T-cell differentiation and activation processes, IL-6 plays an important role by influencing the process, thereby resulting in heightened induction of the adaptive immune system, sustaining chronic inflammation and joint degradation, and significantly contributing to RA-related pathogenesis. These mechanisms also underlie the systemic manifestations of RA, such as pain, fatigue, morning stiffness, anemia, and weight loss, ultimately culminating in bone and cartilage erosion. This change is accompanied by synovial membrane inflammation and thickening, resulting in irreversible joint damage (Takeuchi et al., 2021; Alivernini et al., 2022). Nonetheless, IL-6R blockade has emerged as a crucial target for cytokine blockade in RA treatment, and effective management strategies for treating this disease significantly mitigate the impact of IL-6. Therapeutic approaches for treating RA aim to impede disease progression and prevent joint deformities, and IL-6R blockade represents a targeted treatment strategy. The benefits of IL-6R blockade include improvements in many extra-articular manifestations, including pain, fatigue, and anemia. Furthermore, it may have potentially positive effects on certain comorbidities (Favalli, 2020). Among the innovative treatments for RA and its associated manifestations, within and outside the joint, tocilizumab and sarilumab are biologic drugs approved as humanized IL-6 receptor inhibitors, as recommended in the 2021 treatment guidelines from the American College of Rheumatology (Fraenkel et al., 2021).

Furthermore, herbal medicine research offers promising adjunctive therapies to improve the quality of life of individuals with RA. These include the significant effectiveness of herbal remedies in alleviating RA symptoms, reducing disease manifestations, lowering disease activity scores, minimizing serum acute-phase reactants and RA biomarkers, and decreasing the likelihood of adverse events associated with disease-modifying antirheumatic drugs (DMARDs) (Soeken et al., 2003; Ernst and Posadzki, 2011; Daily et al., 2016; Aryaeian et al., 2019; Li and Zhang, 2020; Zhang et al., 2021; Zeng et al., 2022). A nationwide population-based study conducted in Taiwan revealed a high prevalence of RA patients opting for traditional Chinese medicine (TCM) herbal treatments as complementary therapies. This preference was particularly prominent among patients dealing with anxiety, depression, allergic rhinitis, osteoporosis, menstrual disorders, and menopausal syndrome (Huang et al., 2015).

One prospective candidate, *Piper sarmentosum (PS)*, a member of the *Piperaceae* family, is widely cultivated across the Americas and Asian countries. This versatile plant encompasses roots, dried aerial parts, fruits, and leaves for medicinal applications (Hussain et al., 2012; Sun et al., 2020). However, the aromatic leaves of Southeast Asian nations such as Vietnam, Thailand, Indonesia, and Malaysia have been harnessed for both medicinal and nutritional purposes. For centuries, *PS* has been used as a staple in folk medicine for alleviating symptoms such as fever, rheumatism, arthralgia, diarrhea, dysentery, gastrointestinal disorders, gastritis, toothaches, hyperglycemia, and traumatic injuries (Chi, 1997; Do Tat, 2004; Chaveerach et al., 2006; Hussain et al., 2012; Sun et al., 2020). Remarkably, during the COVID-19 pandemic, *PS* emerged as a commonly used herb for treating various common ailments (Nguyen et al., 2021). Over 140 chemical compounds, including essential oils, alkaloids, flavonoids, lignans, and steroids, have been isolated and identified in *PS* (Sun et al., 2020). The leaves and aerial parts of the plant have been particularly significant in these compound discoveries. Several pharmacological agents, including antiosteoporosis (Suhana Mohd Ramli et al., 2012; Ekeuku et al., 2023; Zainol Abidin et al., 2023), anti-inflammatory agents (Zakaria et al., 2010; Makchuchit et al., 2017; Akmal et al., 2022), antioxidants (Ugusman et al., 2011; Wang et al., 2017), antimalarial agents (Najib Nik A Rahman et al., 1999), antimicrobial agents (Masuda et al., 1991), antihypertensive agents (Mohd Zainudin et al., 2015; Othman et al., 2022), antidiabetic agents (Othman et al., 2022; Nguyen et al., 2023), anticancer agents (Zainal Ariffin et al., 2009; Hematpoor et al., 2018) and neuroprotective (Ridtitid et al., 1998; Yeo et al., 2018)agents, have been found.

Considering both the plant’s safety profile and its longstanding reputation as a *PS* for managing inflammation-associated arthralgia and as a potential osteogenic agent. Flavonoids are recognized as key components in traditional herbal medicine for RA treatment, and our study aimed to assess potential flavonoids present in *PS* leaves through methanol extraction from previous studies (Miean and Mohamed, 2001; Vimala et al., 2003; Ugusman et al., 2012; Purba et al., 2021; Ware et al., 2023). This assessment was performed using computational methodologies, including molecular docking and dynamic simulations. Molecular docking and simulation are important for understanding the interactions between target protein molecules and prospective drug candidates, providing valuable insights into the mechanism of drug activity at the binding site of target proteins (Ekins et al., 2007). To the best of our knowledge, there has been no prior research on the molecular docking and dynamic simulation of bioactive compounds from *PS*. Therefore, our study endeavors to elucidate the molecular-level interaction mechanisms involved and identify potential flavonoid compounds as IL-6 inhibitors of *PS*. This research contributes to our understanding of novel drug opportunities for impeding the local and systemic progression of rheumatoid arthritis.

## 2 Materials and methods

### 2.1 Protein preparation

The Protein Data Bank (PDB:1P9M) repository was used to retrieve the three-dimensional (3D) X-ray crystal structure of the interleukin-6 (IL-6) receptor, followed by removal of the co-crystallized ligands. Next, Glide’s protein preparation wizard panel (Schrödinger Suite 2022–3) was notified for assigning bond ordering, adding hydrogen, or forming disulfide bonds, while Prime was used to fill in missing side chains and loops. OPLS2005 was used to reduce the structure, after which the structure was optimized using PROPKA after water molecules beyond 3.0 of the heteroatoms were eliminated (Ekins et al., 2007; Umar et al., 2021). Finally, the receptor grid file was created to define the ligand-binding pocket.

### 2.2 Ligand preparation

The LigPrep module from the Schrödinger Suite 2022–3 was used to prepare a total of fifty-five (55) phytocompounds of *PS* leaf fractionated extract identified from ethnobotanical databases for molecular docking. The 3D structures were created at low energy with the proper chiralities. At a physiological pH of 7.2 ± 0.2, possible ionization states for every ligand structure were generated. The stereo-isomerities of each ligand were calculated by keeping certain chiralities constant while varying each other (Ojo et al., 2021).

### 2.3 Receptor grid generation

For ligand docking, receptor grid generation allows determination of the position and size of the protein’s active region. Using the receptor grid construction tool in Schrödinger Maestro 12.5, the scoring grid was supported by the crystal structure of interleukin-6 (IL-6). The van der Waals (vdW) radius scaling factor of the nonpolar receptor atoms was set to 1.0, with a partial charge cutoff of 0.25. Consequently, the top-scoring compound from the molecular docking analysis was further subjected to induced fit docking using Maestro 12.5’s induced fit docking panel.

### 2.4 Protein–ligand docking

The outcome of the receptor grid file was utilized to perform molecular docking investigations with the Glide tool of Schrödinger Maestro 12.5. Standard precision (SP) was employed to dock the protein interleukin-6 (IL-6), and therefore, ligands were prepared from *P. sarmentosum Roxb* along with the quality activator, keeping the ligand sampling set to flexible and ligand sampling set to none (refine only). For ligand atoms, the vdW radius scaling factor was scaled at 0.80 with a partial charge cutoff of 0.15.

### 2.5 Multiple-ligand and receptor–ligand complex modeling

The chemistry of the ligands was properly standardized and extrapolated, and the structure files (SDF) of the test compounds sourced from the PubChem database were prepared using the LigPrep panel of the Schrödinger suite (Schrödinger 2022–3, LLC, New York, NY, United States) and were used for modeling via PHASE. PHASE automatically aligns the ligands and supports the optimum arrangement and mutual characteristics of the ligands. PHASE was employed to create a receptor–ligand complex model employing the highest nine compounds with the best binding affinity against the target protein regarding the quality. The hypothesis was set with a maximum number of features to be created as 7.00, a minimum feature-feature distance of 2.00, and a minimum feature-feature distance of 4.00 for components of the identical type and donors as vectors. Aside from the donor and negative ionic features, which were set to 1, the hypothesis difference criteria were retained, and the acceptor and negative features were made equivalent.

### 2.6 Binding free energy calculation

The Prime Molecular Mechanics-Generalized Born area MM-GBSA tool (Schrödinger suite 2022–3) was used to determine the stability of protein–ligand complexes according to their binding free energy. The ligands were prepared beforehand using LigPrep, and therefore, the relevant proteins were prepared using the protein preparation wizard, as described previously. Sitemap anticipated the active sites of the proteins. Glide standard precision (SP) docking was then performed to dock the chemicals with proteins. The MM-GBSA technology offered with Prime was utilized to determine the binding free energy for ligand‒protein complexes utilizing the Prime MM-GBSA panel. The OPLS3 physical phenomenon was chosen; therefore, the continuum solvent model was VSGB. The default settings for the opposite options were selected (Ojo et al., 2021). The concentration of each of the ligands containing ΔGbind with the LASV nucleoprotein was calculated via the following equation: ΔGbind = ΔE + ΔGsolv + ΔGSA (1) ΔE = E_complex_ - E_protein_–E_ligand,_ where E_complex_, Eprotein and E_ligand_ are the minimized energies of the protein–inhibitor complex, protein, and inhibitor, respectively. Furthermore, ΔGsolv = Gsolv (_complex_) - Gsolv (protein) - Gsolv (_ligand_), where Gsolv (_complex_), Gsolv (_protein_), and Gsolv (_ligand_) contain the solvation free energies of the complex, protein, and inhibitor, respectively. ΔGSA = GSA (_complex_) - GSA (_protein_) - GSA (_ligand_), where GSA (_complex_), GSA (_protein_), and GSA (_ligand_) serve as the surface area energies for the complex, protein, and inhibitor, respectively (Ojo et al., 2021).

### 2.7 Pharmacological parameters

The absorption, distribution, metabolism, excretion, and toxicity (ADMET) of the test drugs were assessed using *in silico* integrative model predictions from the SwissADME and PROTOX-II servers.

### 2.8 Molecular dynamics simulations and trajectory point analysis

MD simulations of the receptor of interest (1P9M) were performed using Schrödinger Suite 21.3 with Maestro version 12.5.137, MM share version 5.7.137, and Windows-x64 Platform. The construction of the MD preparation and trajectory analysis methods followed previous similar methods (Adekiya et al., 2022). The docked complexes were subjected to molecular simulation following the Desmond module of the Schrödinger software with an OPLS 2005 force field. The protein–ligand complex was bounded with a predefined transferable intermolecular potential with a three-point water model in an orthorhombic box. The neutralization of the overall charge was minimized by the addition of sodium and chloride ions to mimic physiological conditions. Both the temperature and pressure were kept constant at 310°C and 1.01325 bar, respectively, by using a Nose‒Hoover thermostat and a Martyna-Tobias-Klein barostat made from the United States. The simulation relaxation was undertaken by using an NPT ensemble after considering the number of atoms, the pressure, and the timescale. During the MD simulation, the long-range electrostatic interactions were calculated by using the particle mesh Ewald method. Furthermore, MD simulation analysis was carried out for 100 ns, and trajectory sampling was set at an interval of 100 ps with 1000 frames. The simulation outputs were analyzed and visualized by a simulation interaction diagram and an MS-MD trajectory analysis. The MD analysis was performed in replicate to avoid variation. The data was plotted by using OriginPro version 9.

## 3 Results

**FIGURE 1:**
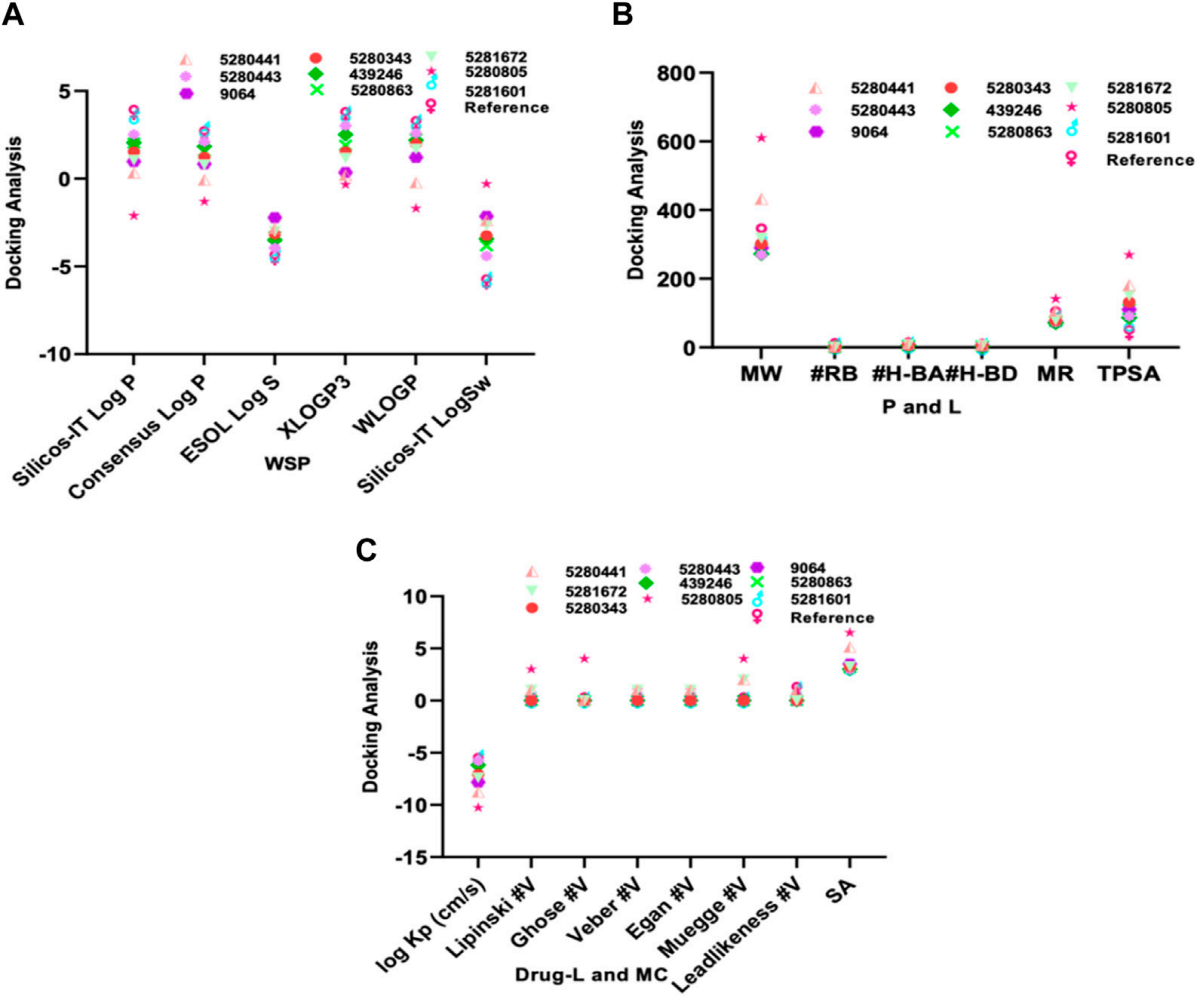
The ADEMET predictive study of **(A)** WSP water soluble properties, **(B)** pharmacokinetics, lipophilicity, **(C)** drug likeness and medicinal chemistry of the top-scoring compounds from *PS* leaf fractionated extract.

In molecular docking analysis, the significance of the structure of each molecule identified from a medicinal plant is crucial when compared to established molecules due to their molecular interactions, which may be influenced by the solute state of the water molecule in the aqueous phase. Therefore, ADEMET prediction studies serve as vital bioinformatics tools for *in silico* analysis, where toxicity predictions for the chosen compounds indicate their relative safety, as illustrated in Figure 1. Moreover, the potential of all the identified agents as small molecules when acknowledging their druggable potency can be easily observed in the active transport system of cells; however, the standards for drug likeness do not apply to natural bioactive substances. Therefore, the identified compounds exhibited therapeutic potential via protein binding and the rift domain in the State one protein complex, which revealed a small number of ligands with outstanding results, as shown in Table 1, with no breaches of Lipinski’s rule of five in the categories of compounds identified, as shown in Figures 1A, B. The *in silico* analysis of the binding free energy potential revealed that the predicted molecules had greater free binding energies than did the reference molecules, as shown in Table 1. Amazingly, based on the results obtained with the Qikprop Schrödinger suite version from Figure 1C, the ligands of interest did not inhibit Pgp, suggesting that they favorably compete with reference small molecules predicted to be inhibitors of IL-6 in Pgp. Moreover, in comparison to reference small molecules, the ligands derived from the fractionated extract of PS leaves demonstrated gastrointestinal absorption. Consequently, a majority of these ligands function as ATP-dependent drug efflux pumps for ADME compounds with significant potency due to their substrate specificity, as indicated in Table 2.

**TABLE 1:** MM-GBSA binding free energy (∆G) calculations and binding sites of the top-scoring proteins from the *Piper sarmentosum* Roxb leaf fractionated extract.

ID	P-MMGBSA	ROF	ROT	MMGBSA	H-bond	Others
5280441	−9.96	1	2	−44.17	ASN60, LEU57	2 (ARG168), LYS56
5281672	−9.60	1	1	−39.73	MET67	2 (LYS66)
5280343	−7.47	0	1	−30.59	MET67	LYS66
5280443	−10.66	0	0	−42.62	LEU62, LYS66	0
139246	−9.07	0	0	−36.25	ASN66, LYS66, LUE62, GLU172	ARG168
5280805	−6.99	3	2	−33.31	2 (ASN61), LYS66, 2 (ALA58)	2 (LYS66)
9064	−8.83	0	1	−35.72	CYS73, ARG179, 2 (GLU172)	0
5280863	−8.84	0	0	−35.79	LYS66, LEU62, ASN60, GLU172	2 (ARG168)
5281601	−9.46	0	0	−38.74	ASN 60	ARG168, ASN66
2353	−8.77	0	0	−37.03	0	ARG179

Legend: MM-GBSA: Molecular Mechanics-Generalized Born area; P-MMGBSA: Prime Molecular Mechanics-Generalized Born area; ROF: rule of five; ROT: rule of three; (5280441: Vitexin; 5281672: Myricetin; 5280343: Quercetin; 5280443: Apigenin; 139246: Naringenin; 5280805: Rutin; 9064: Catechin; 5280863: Kaempferol; 5281601: 5-hydroxy-7, 4-dimethoxyflavone; Reference: Berberine.

**TABLE 2:** Pharmacokinetic prediction of the five top-scoring compounds from the fractionated *Piper sarmentosum* Roxb leaf extract.

GI-A	BBB-P	Pgp-S	CYP1A2-I	CYP2D6-I	CYP3A4-I
Low	No	No	No	No	No
Low	No	No	Yes	No	Yes
High	No	No	Yes	Yes	Yes
High	No	No	Yes	Yes	Yes
High	No	Yes	Yes	No	Yes
Low	No	Yes	No	No	No
High	No	Yes	No	No	No
High	No	No	Yes	Yes	Yes
High	Yes	No	Yes	Yes	Yes
High	Yes	Yes	Yes	Yes	Yes

Note: GI, gastrointestinal tract; BBB, blood brain barrier, Pgp = P-glycoprotein, CYP , Cytochrome P450 family.

Furthermore, our *in silico* analysis of drug likeness and ADMET properties revealed that all the compounds (except vitexin, myricetin, and rutin) identified from the fractionated extract of *P. sarmentosum Roxb* leaf are drug-like candidates based on the Lipinski rules of drug-like small molecules. In addition, the synthetic accessibility of any drug-like compound is very important in many areas of drug discovery. Moreover, historical knowledge of synthetic compounds analyses the information obtained from millions of previously synthesized chemicals and considers the complexity of the molecules. Therefore, the synthetic products obtained from our Insilco platform compete favorably with the reference molecule, as shown in Figure 1C.

Furthermore, one of the compounds identified, 5-hydroxy-7,4-dimethoxyflavone, has the potential to compete with reference molecules with high gastrointestinal absorption and the ability to cross the blood–brain barrier (BBB) (Table 2).

## 4 Discussion

Despite the surge of several diseases worldwide, the use of bioactive molecules from medicinal plants for the treatment and management of diseases is still important and has received increased attention in recent decades (Udenze et al., 2014; Onikanni et al., 2022). *PS* plants, including leaf parts, are known for their medicinal value in the treatment and management of human diseases, apart from their traditional famous role in Chinese medicine, as indicated in the Supplementary Appendix of the Chinese pharmacopoeia because of their medicinal importance and edible value (Sun et al., 2020; Xu et al., 2021). In addition to its nutritional value, research has revealed its higher level of consumption as food due to its high proportion of proteins, minerals and fatty acids, which help yin and regulate endocrine functions (Yeoh and Wong, 1993). Moreover, the rise in the prevalence of various debilitating illnesses globally necessitates further investigation. Cardiovascular disease and inflammatory autoimmune conditions, such as rheumatoid arthritis, are prime examples. Rheumatoid arthritis is a significant condition that triggers joint inflammation, leading to exhaustion, anemia, and osteoporosis in the body (Dayer and Choy, 2010).

Moreover, as one of the major important receptors, IL-6 plays a role as a complex whole by combining with the host defense against any attack agent of infection and damage to tissues via the ability to induce acute phase reactions and blood cell production, which also has devastating effects due to its uncontrolled increase in IL-6 production, thereby resulting in different immune-mediated developmental inflammation and diseases (McInnes and Schett, 2007; Yoshida and Tanaka, 2014). However, additional information needs to be obtained on the anti-rheumatoid arthritis action of individual phytocompounds of *PS* leaf for further development of novel drugs for the treatment and management of rheumatoid arthritis. Utilizing a target-based computational approach, ligands extracted from PS leaves were screened for potential antirheumatoid arthritis activity by modeling the interaction between a receptor or protein target and a drug candidate (Sim et al., 2009; Sun et al., 2020). This approach is also useful for assessing the binding affinities of ligands for proteins, thereby enabling the depiction of the behavior of a drug candidate within the binding cavity of a receptor and hence providing insight into the biological function of the drug. Interestingly, our molecular docking of the identified compounds from the fractionated extract of *P. sarmentosum Roxb* leaf revealed therapeutic potential via protein binding and the rift domain in the State one protein complex, with a small number of ligands showing outstanding results, as shown in Figure 1.

Furthermore, the BBB is a limiting factor in the therapeutic effects of most medications for several disorders (Onikanni et al., 2022). Interestingly, our druggable candidate exhibited high bioavailability (0.85:85%) and was permeable to the BBB, suggesting its suitability for the treatment of neuronal impairment. Therefore, our study provided preclinical evidence that the fractionated extract of *P. sarmentosum Roxb* leaf attenuates rheumatoid arthritis complications via the inhibition of IL-6 influx. In addition, based on the molecular docking analysis from the *in silico* study, the binding energies of the five top-scoring phytocompounds were compared favorably with that of the reference ligand given that one of the promising compounds (5-hydroxy-7,4-dimethoxyflavone) is a potent inhibitor of IL-6 with a binding potential energy of −9.46 compared with the reference ligand (−8.77), as shown in Table 1. Hence, this finding provided a better indication that this fractionated extract of *P. sarmentosum Roxb* leaf compound could inhibit the activity of IL-6 as a reference ligand.

Moreover, historical information on synthetic compounds has been obtained from millions of already synthesized chemicals, and the complexity of the molecules has been considered. Therefore, the synthetic products obtained from our *in silico* compete favorably with the reference molecule, as shown in Figure 1. Therefore, one of the compounds identified from the fractionated extract of *P. sarmentosum Roxb* leaf (5-hydroxy-7,4-dimethoxy flavone) strongly competes with the reference molecule because of its high gastrointestinal absorption and ability to cross the blood–brain barrier (BBB), as shown in Table 2. According to previous studies, the polypeptide chain is composed of 212 amino acid residues that are folded into different domains of unequal size with a 28-amino acid signal peptide, and its gene maps to chromosome 7p21. The Molecular Mechanics-Generalized Born Expanse (MM-GBSA) was also used to validate the results of the molecular docking analysis. Furthermore, this analysis revealed how the binding affinity score can predict the specificity and binding strength of a ligand in the binding pocket of a receptor (Coleman, 2011; Genheden and Ryde, 2015; Zainab et al., 2020). Therefore, the results of our docking analysis of the complex hexameric human IL-6 of the alpha receptor with 5-hydroxy-7,4-dimethoxy flavone strongly correlated with the prime molecular mechanics-generalized band area −38.74. Compared with the reference ligand of −37.03, which produced unique hydrogen bonds, ASN60 was found to reside against the reference ligands with salt bridges containing ARG168 and ASN66 of the identified compound against the reference ligand of the ARG179 amino acid residue, as shown in Table 1.

**FIGURE 2:**
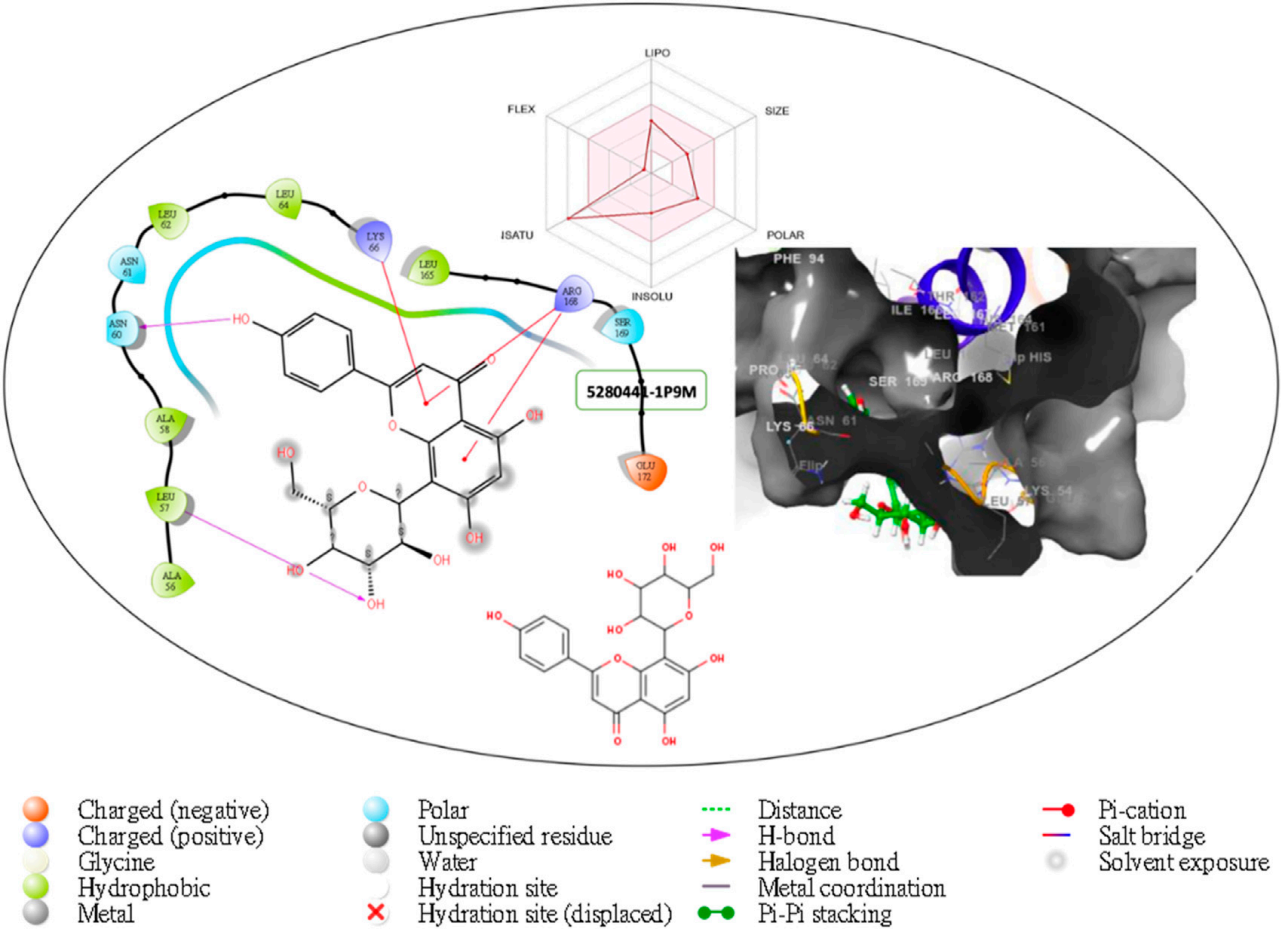
Comprehensive 2D and 3D molecular interaction data for 5280441 with the receptor (interleukin-6 (IL-6)) obtained from docking analysis.

**FIGURE 3:**
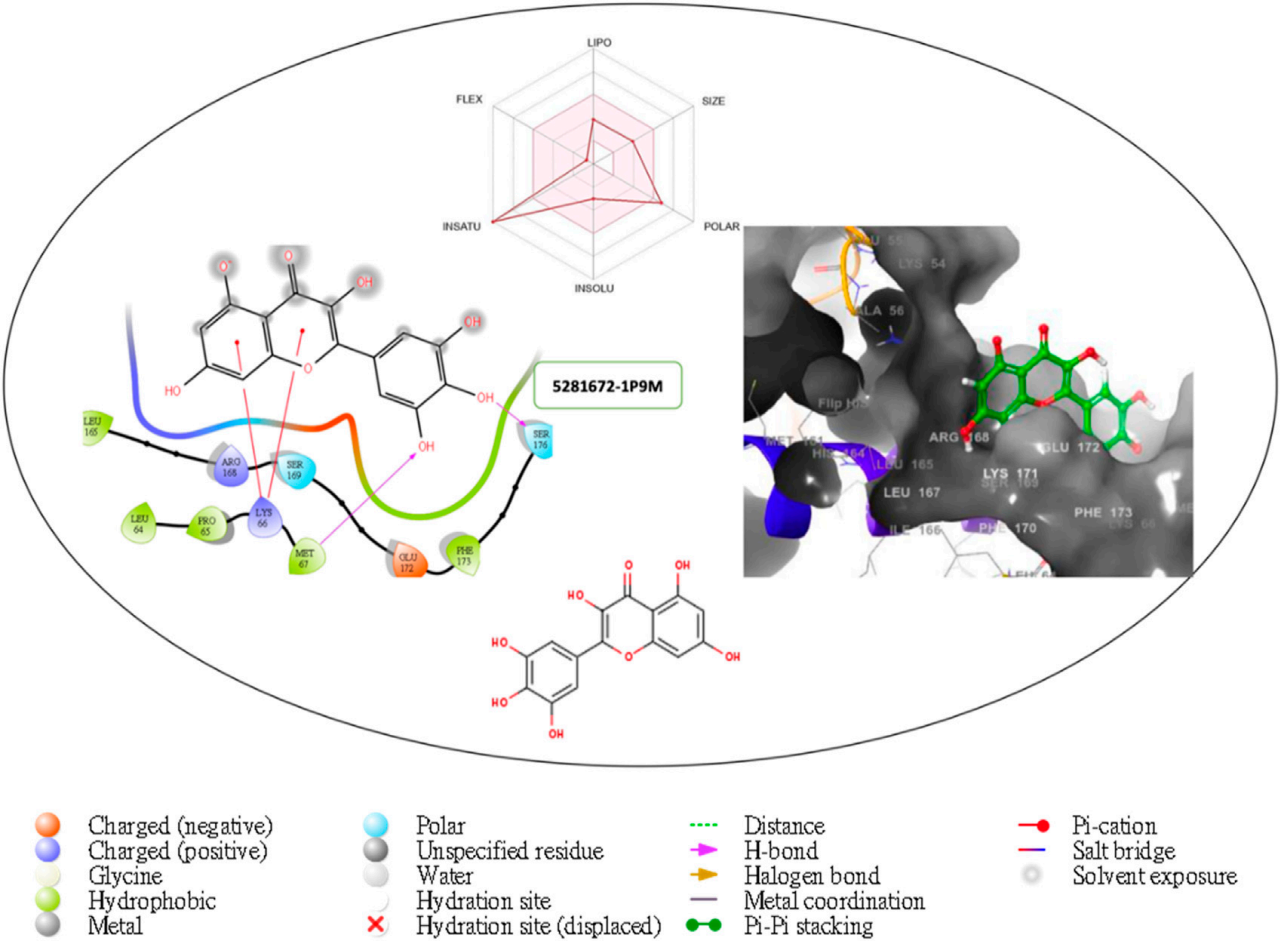
Comprehensive 2D and 3D molecular interaction data for 5281672 with the receptor (interleukin-6 (IL-6)) obtained by docking analysis.

**FIGURE 4:**
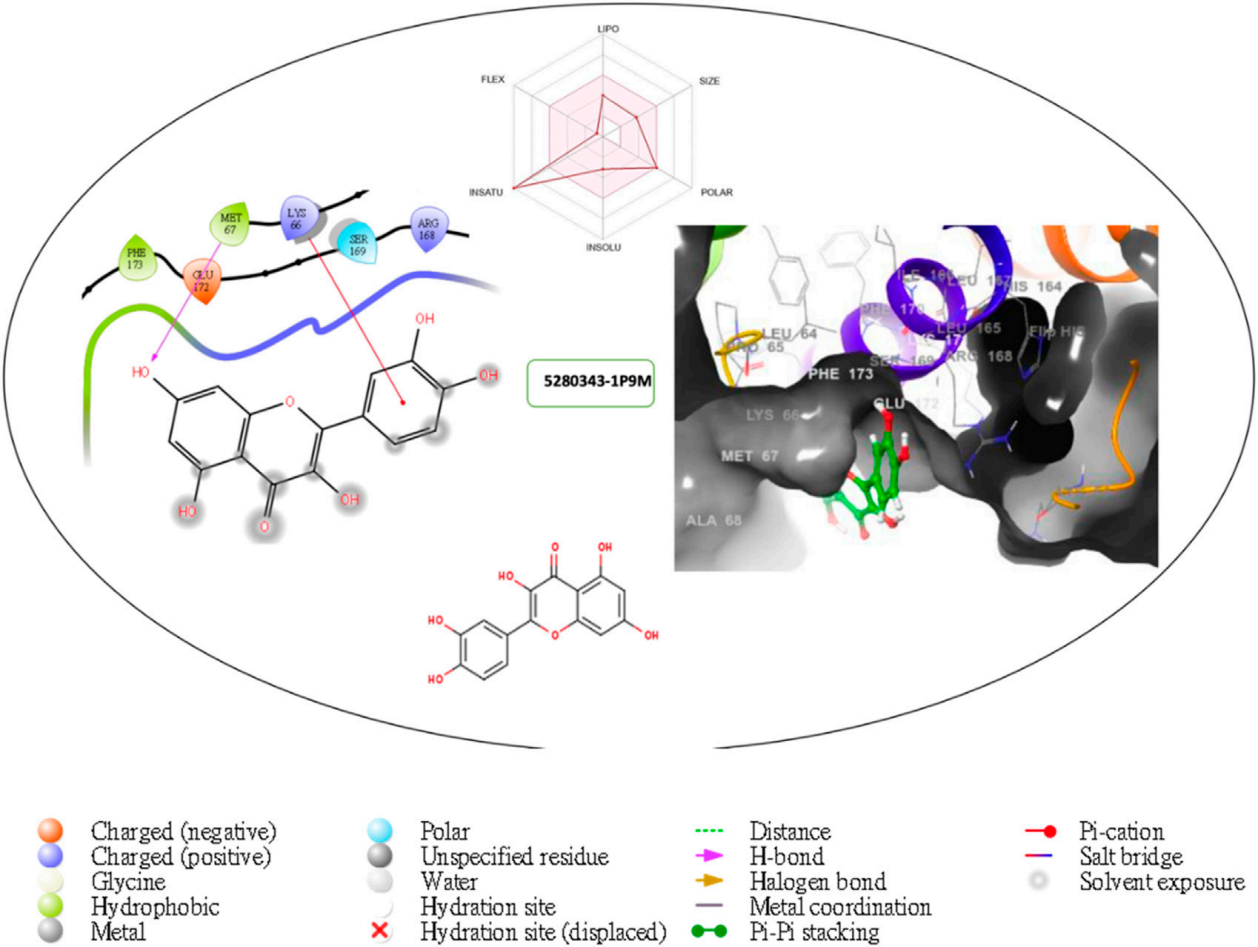
Comprehensive 2D and 3D molecular interaction packages of 5280343 with the receptor (interleukin-6 (IL-6)) from docking analysis.

**FIGURE 5:**
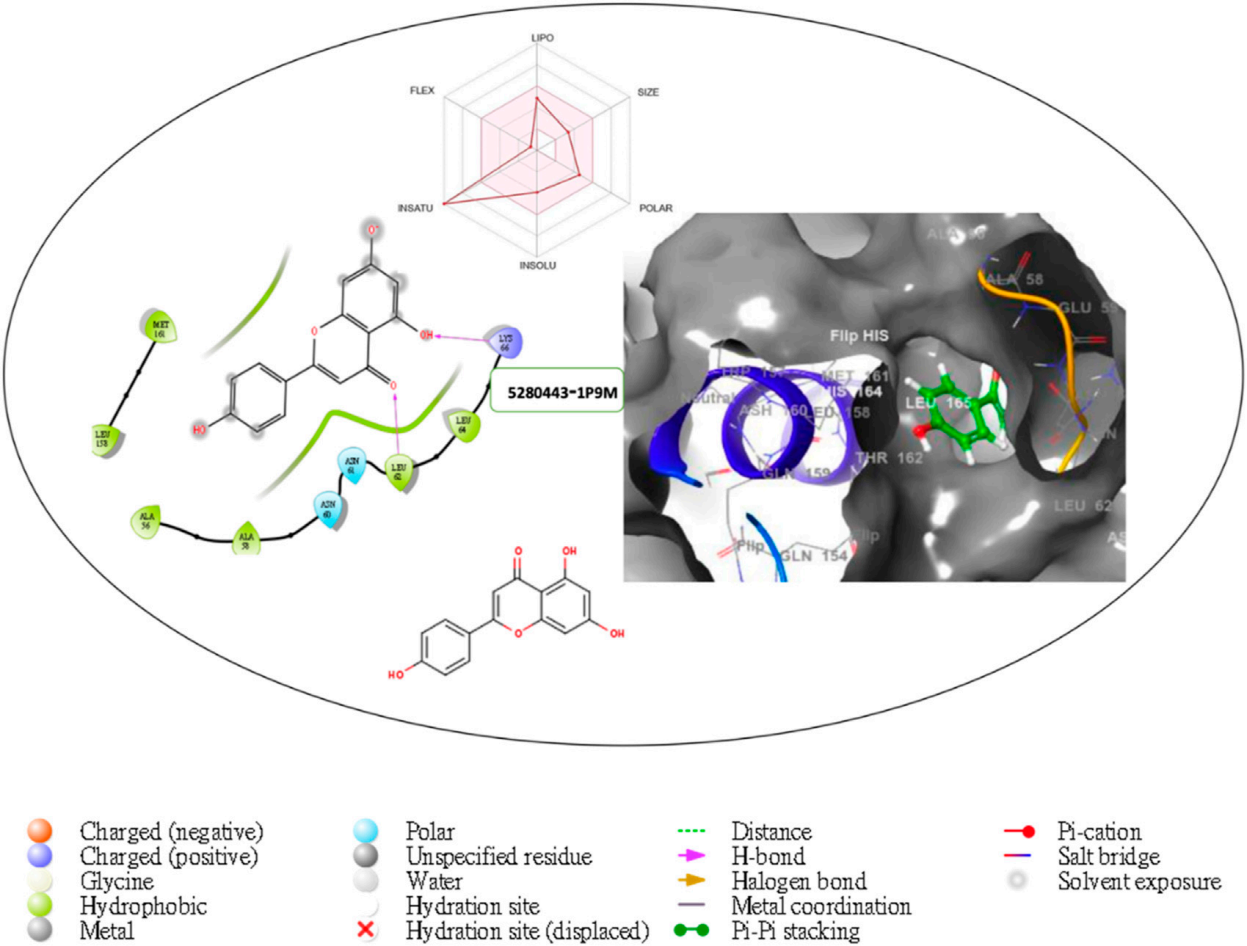
Comprehensive 2D and 3D molecular interaction packages of 5280443 with the receptor (Interleukin-6 (IL-6)) from docking analysis.

**FIGURE 6:**
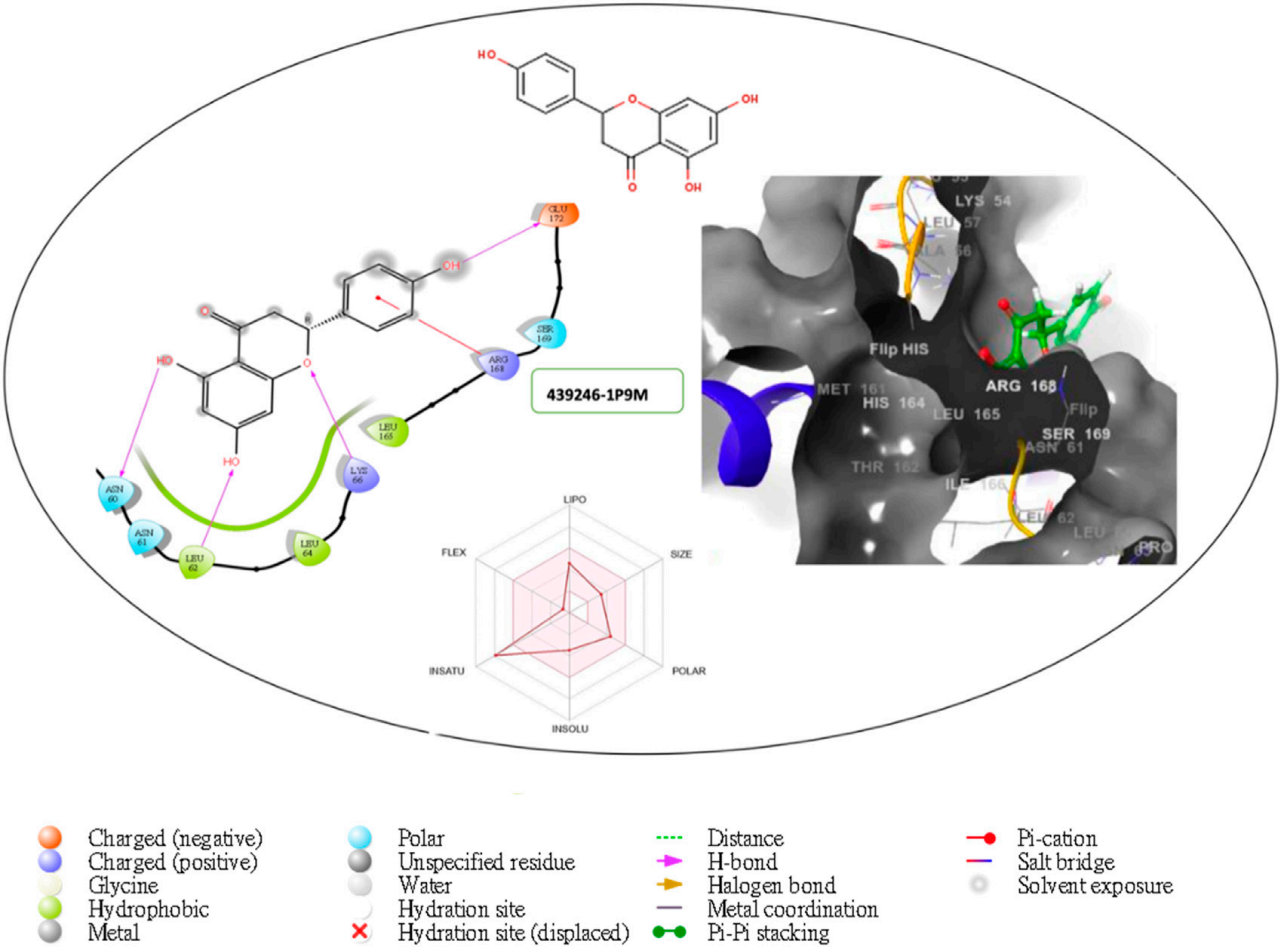
Comprehensive 2D and 3D molecular interaction packages of 439246 with the receptor (interleukin-6 (IL-6)) from docking analysis.

**FIGURE 7:**
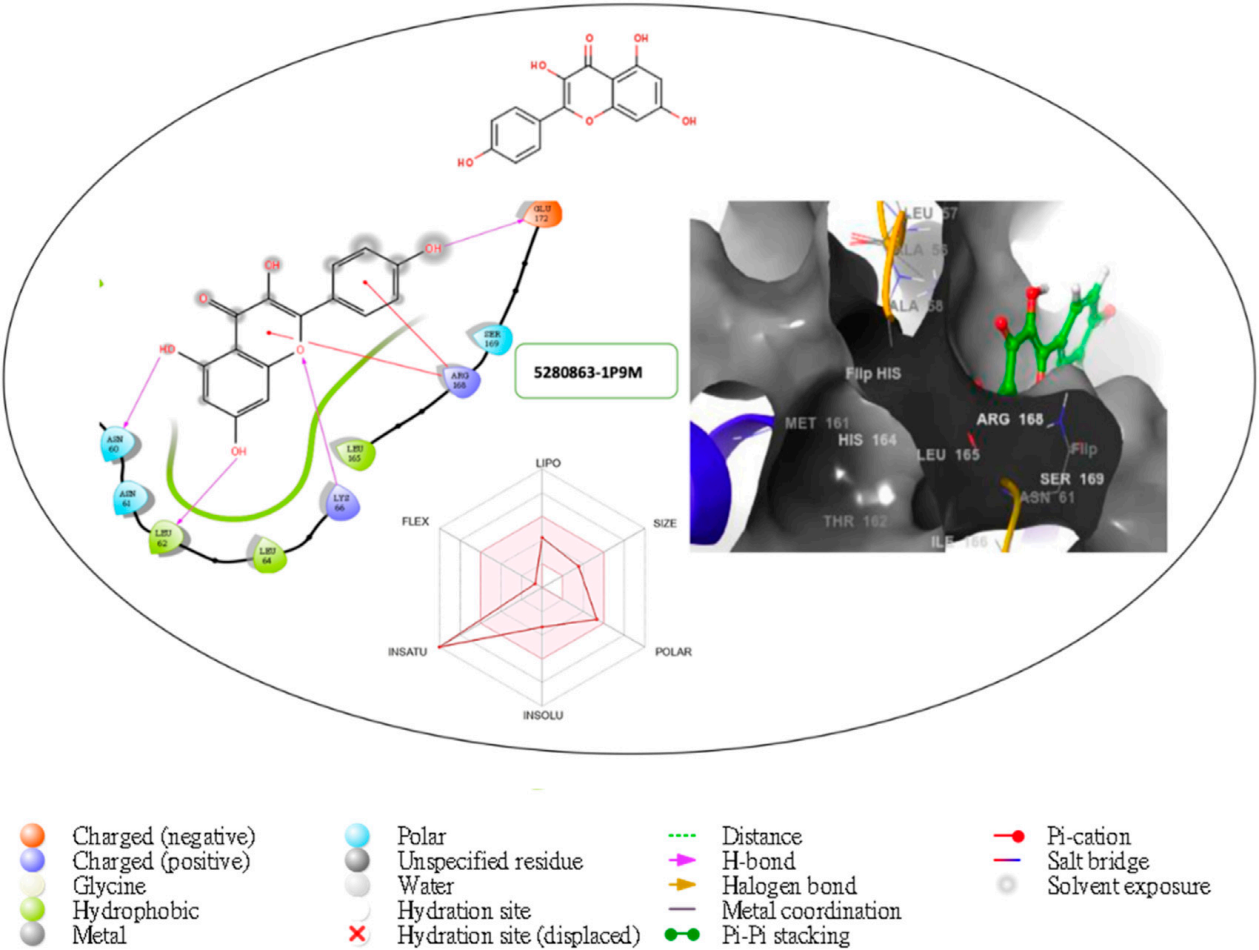
Comprehensive 2D and 3D molecular interaction packages of 5280863 with the receptor (interleukin-6 (IL-6)) from the docking analysis.

**FIGURE 8:**
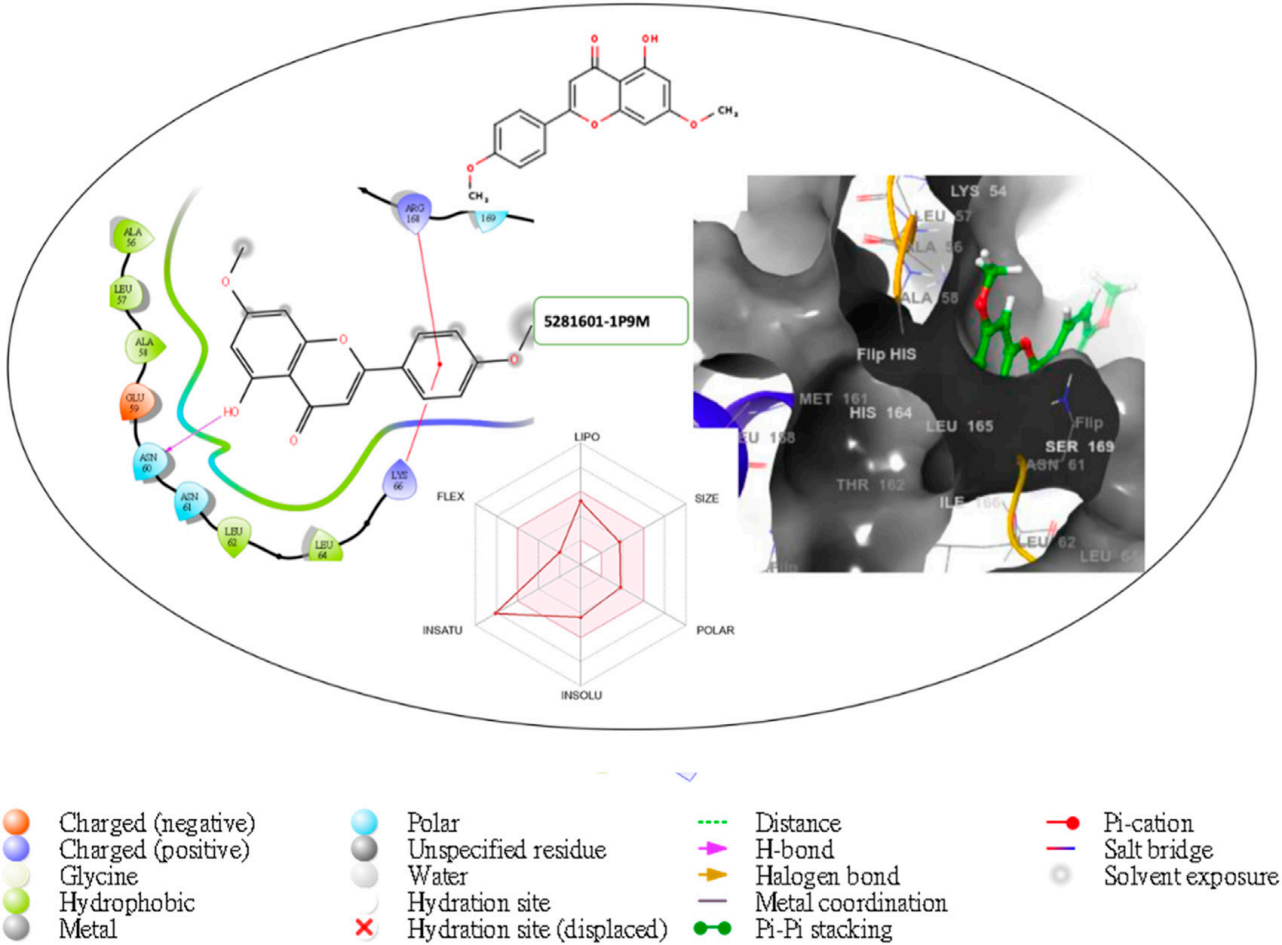
Comprehensive 2D and 3D molecular interaction packages of 5281601 with the receptor (interleukin-6 (IL-6)) from docking analysis.

**FIGURE 9:**
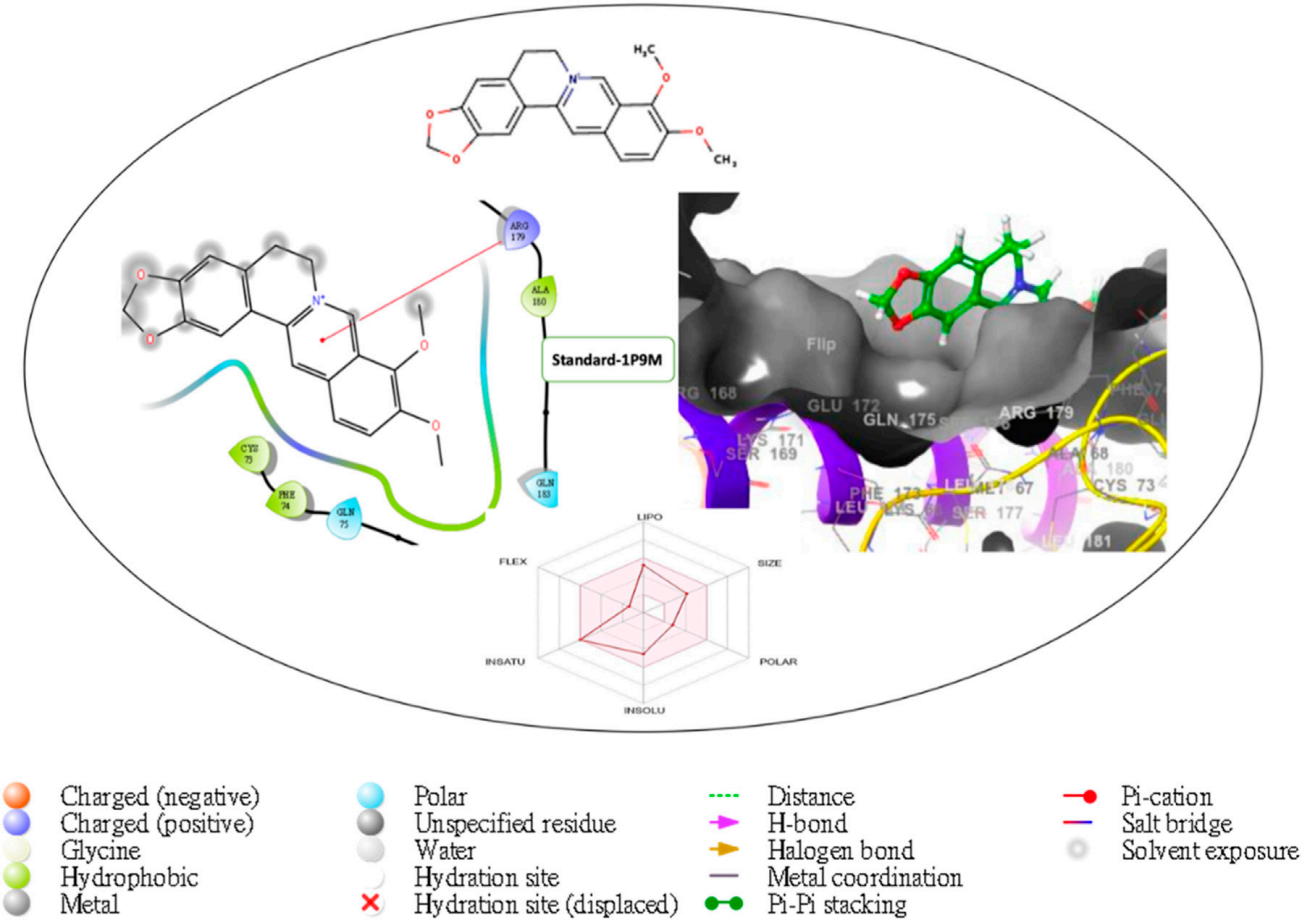
Comprehensive 2D and 3D molecular interaction packages of standard with the receptor (Interleukin-6 (IL-6)) from the docking analysis.

**FIGURE 10:**
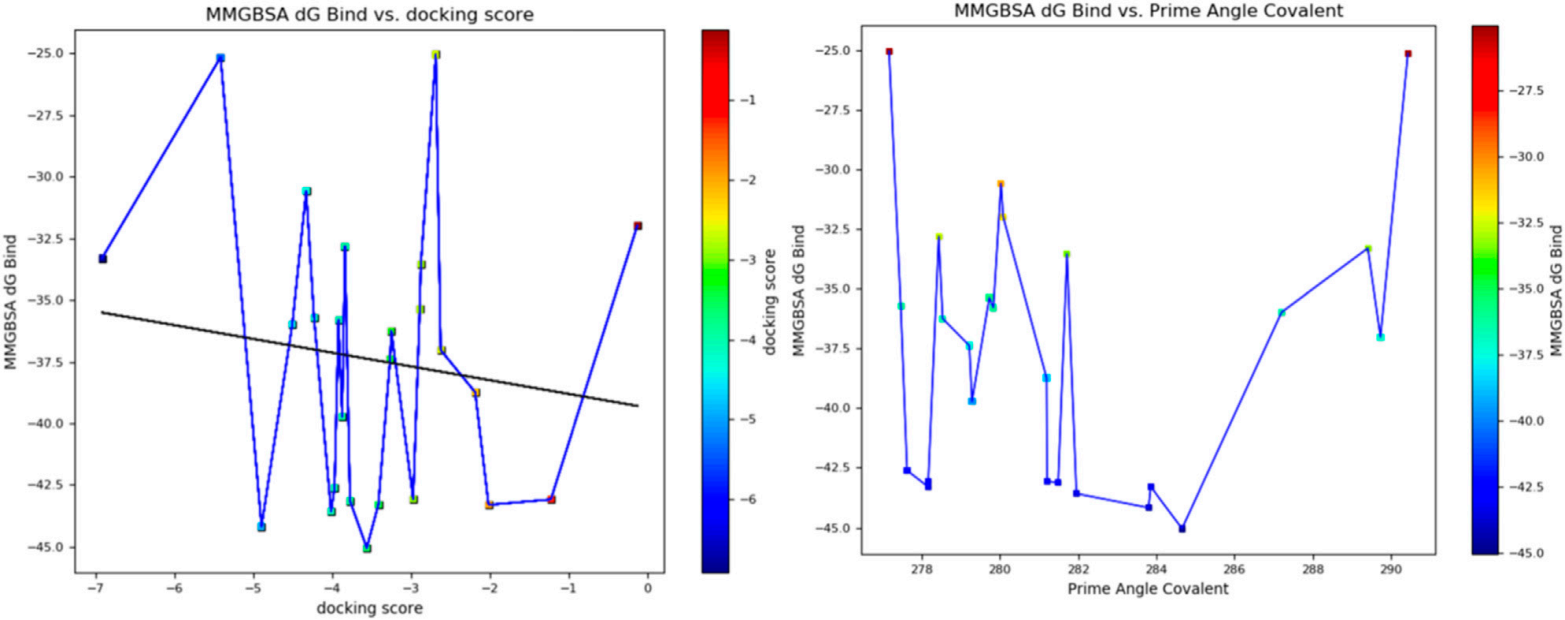
Plot of free binding energy against docking score from *in silico* analysis of the ligands identified from the fractionated *Piper sarmentosum* Roxb leaf extract.

Furthermore, the structural interaction revealed in Figures 3–10 observed similar event with Apigenin when docked with hexameric human IL-6 of alpha receptor having displayed - Prime Molecular Mechanics-Generalized Born area −10.66 and Molecular Mechanics-Generalized Born area −42.62, respectively shown in Table 1 with hydrogen bonds of LEU68 and LYS66 compared with against the reference ligand of ARG179 amino acid residue. However, compared with those of the reference ligands, the intermolecular interactions with the residues revealed strong interactions, as shown in Table 1, and the structural interactions are displayed in Figures 2–9. In addition, the *in silico* investigation also unveiled the covalent priming angle of each ligand by plotting free binding energy against the docking score from the *in silico* analysis of the ligands identified from the PS leaf fractionated extract. This highlighted the potential binding pocket of the promising compound in comparison to the reference ligand, as illustrated in Figure 10, showcasing the interresidue contacts within the binding pocket.

**FIGURE 11:**
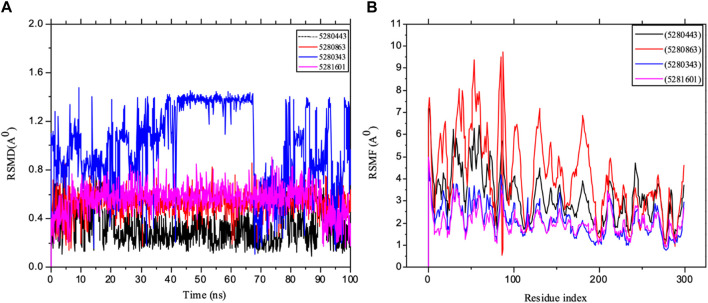
MD simulation of 1P9M complexed with 5280343 (quercetin), 5280443 (apigenin), 5280863 (kaempferol) and 5281601 (5-hydroxy-7,4-dimethoxyflavone). **(A)** L_RMSD and **(B)** P_RMSF graphical illustration plots. All the simulations were carried out using the Schrödinger suite version maestro v21.3.

Furthermore, an important aspect of drug discovery requires molecular simulation, which could play a vital role in any experiment (Durrant and McCammon, 2011; Borhani and Shaw, 2012). Many key drug discovery targets have been revealed by structural biology, allowing us to exploit the power of structure-based drug design, which requires the consideration of the dynamic properties of proteins. Moreover, one of the valuable steps in deciphering the functional mechanism of proteins and other biomolecules in revealing the structural basis of several diseases is molecular simulation, which is crucial in the design and optimization of proteins, small molecules and peptides. Therefore, our *in silico* study captured potential immune protein inhibitors in full atomic detail and at very clear and better temporal resolution. The mean square deviation (RMSD) of the backbone of the target was used to evaluate the stability of the system, thereby measuring the amount of protein change with respect to the initial structure during the simulation period, i.e., the structural distance between the coordinates. The results of the RSMF analysis of the IL-6 targets were carefully studied using 400 ns trajectories, as shown in Figure 11. The importance of protein structure prediction in a competitive assessment based RSMD is a critical point for revealing how the submitted structure fits into the target structure, thereby indicating that a lower RMSD is a better model than is the case for the target structure. The RMSD of the complex in the major system did not deviate drastically over the simulation testing period studied. The RSMD of the system observed mostly in the present study was within the range of 0.3–1.4 Å, indicating that the conformation changed with respect to IL-6. Furthermore, the simulation converged and showed some stability (approximately 50–100 ns) in most of the systems, which revealed some system equilibration and simulatory time that was sufficient for rigorous analysis. The results are also presented as the mean ± SEM of the deviation plot, as shown in Table 3.

**TABLE 3:** Molecular dynamics simulation properties of the native protein and ligands of interest.

Receptor (M)	Ligand	RSMF	RSMD	rGyr	MolSA	SASA	PSA
1P9M	5280443	3.21 ± 1.15	0.32 ± 0.13	3.66 ± 0.22	239.75 ± 1.12	106.47 ± 35.66	194.15 ± 2.03
	5280863	4.14 ± 1.82	0.53 ± 0.09	3.63 ± 0.02	245.15 ± 1.28	155.23 ± 17.25	229.09 ± 2.34
	5280343	2.10 ± 0.73	1.01 ± 0.32	3.74 ± 0.03	253.75 ± 1.37	255.81 ± 45.90	276.16 ± 2.63
	5281601	2.01 ± 0.58	0.56 ± 0.11	4.74 ± 0.03	310.44 ± 1.58	484.56 ± 101.92	57.44 ± 4.02

Note: Values are represented as the mean ± SEM, measured in the Armstrong unit (Å). RMSD: root mean square deviation; RMSF: root mean square fluctuation; rGyr: radius of gyration; MolSA: molecular surface area; SASA: solvent-accessibility surface area; PSA: pressure swing adsorption.

Furthermore, the dynamic behavior of the protein residues was studied by examining the RSMF pattern, as shown in Figure 11. The fluctuations of the complexed molecules are relatively similar and were observed within 1.5–8.5 Å within the stabilized system up to 100 ns of the simulation. The analysis of the mean ± SEM of the plots in the RFs of receptor 5280863 has the highest RSMF value (4.14 ± 1.82), and that of the receptor 5280443 has the highest RMSF value (3.21 ± 1.15), as shown in Table 3.

**FIGURE 12:**
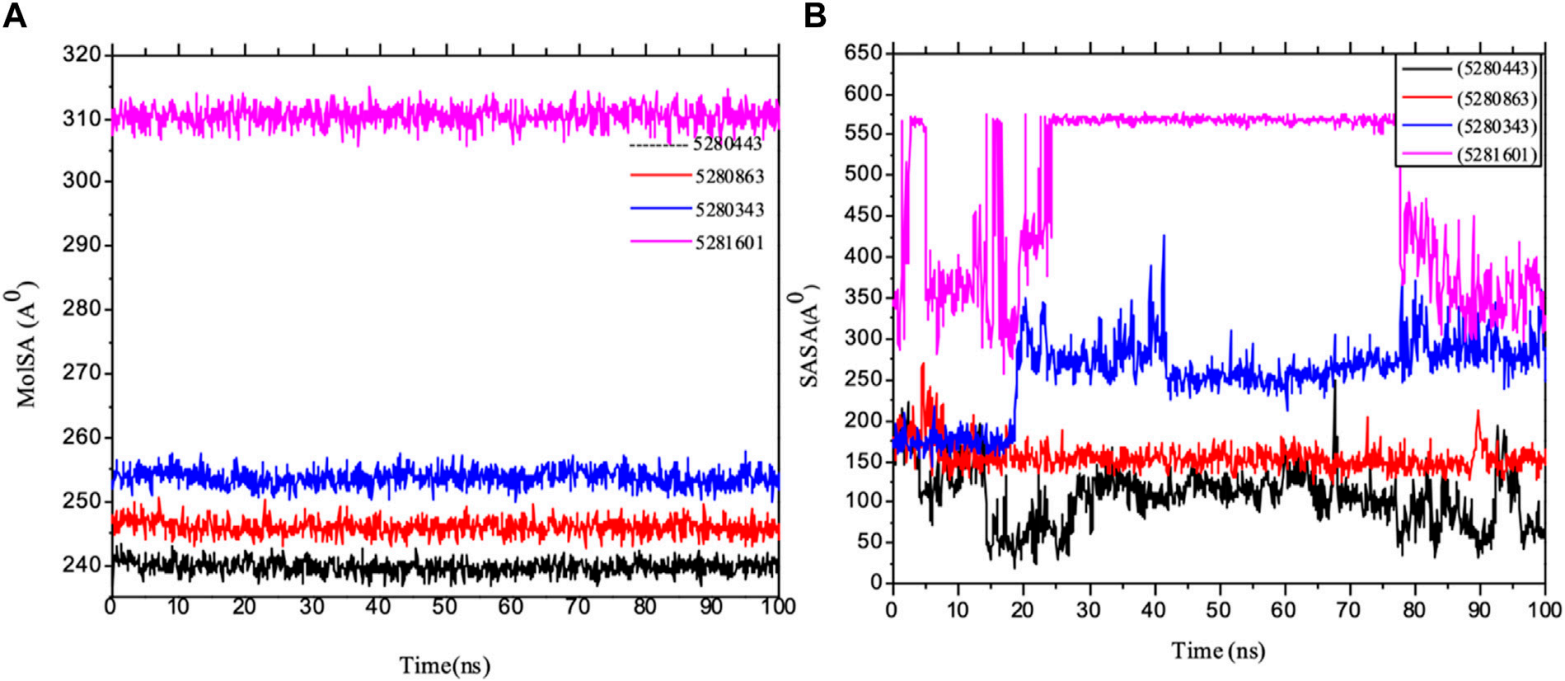
MD simulation of 1P9M complexed with 5280343 (quercetin), 5280443 (apigenin), 5280863 (kaempferol) and 5281601 (5-hydroxy-7,4-dimethoxyflavone). **(A)** MolSA representation and **(B)** SASA diagram. All the simulations were carried out using the Schrödinger suite version maestro v21.3.

**FIGURE 13:**
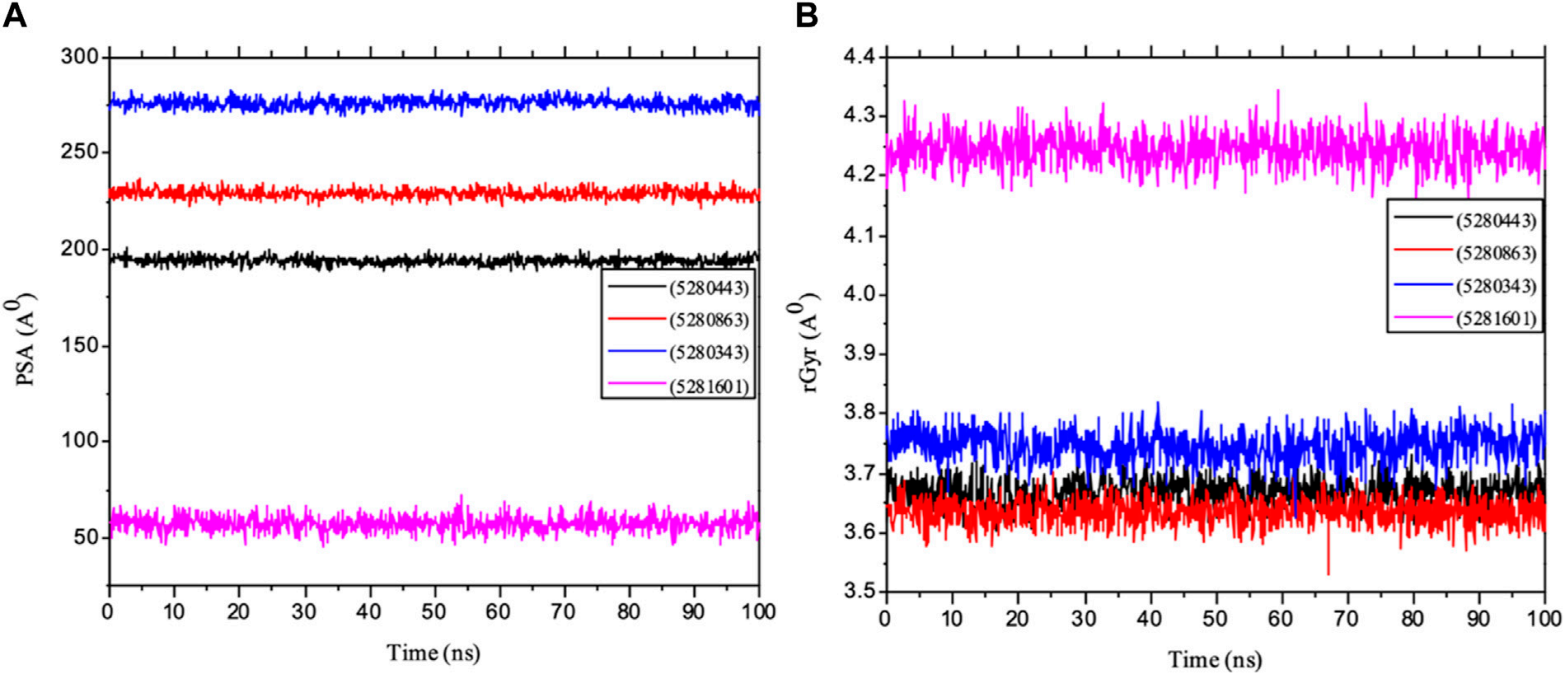
MD simulation of 1P9M complexed with 5280343 (quercetin), 5280443 (apigenin), 5280863 (kaempferol) and 5281601 (5-hydroxy-7,4-dimethoxyflavone). **(A)** rGyr representation and **(B)** PSA diagram. All the simulations were carried out using the Schrödinger suite version maestro v21.3.

Importantly, MolSA fluctuations are mostly associated with the rearrangement of amino acid residues from either the accessible region or the buried region, thereby causing a change in protein–ligand structural modeling. Therefore, our study revealed that the reorganization of amino acid residues from either the available point or the buried point from the ligands to the receptor of interest was relatively similar throughout the simulation period. Both the dynamic behavior of the protein residues and pattern and the differences in the mean deviation were 239.75 ± 1.12, 245.15 ± 1.28, and 253.75 ± 1.37, respectively, as shown in Table 3. Furthermore, the pressure swing adsorption (PSA) analysis of the mean ± SEM of the plots showed similar results for most of the ligands, with deviation plots and a mean ± SEM for the receptor binding complex, as shown in Table 3 and Figure 12, with no noticeable decrease in pattern from the original pattern position until the end of the simulation period. With the strong interactions between IL-6 and its atoms connected with amino acid residues in the binding pockets of said receptor, changes in conformation could be induced, thereby affecting the binding interactions that could lead to receptor inhibition. The stability of the SASA values remains constant when the receptor of interest is folded into a particular conformation. However, the unfolding of this receptor could eventually lead to a larger surface area being exposed to the solvent, thereby indicating that the SASA was folded or unfolded at the position of the receptor of interest. Moreover, the crystal structure of hexameric human IL-6 receptor which examined the interactions between the protein surface and surrounding solvent molecules revealed the event in solvent-accessibility surface area from 50 ns until the end of the simulation period with equilibrium till the end of the simulation pattern as shown in Figure 13. In addition, the compactness of the receptor, perturbations, and folding status in gyration (rGyr) values reveal unfolding events in the structure, revealing higher and lower (rGyr) representations. The radius of gyration was analyzed to determine changes in compactness. The mean plot shows a strongly stabilized system with the complex from 10 ns of the simulation period time throughout the 100 ns simulation period, as shown in Figure 13, with the simulation converging and showing some stability at approximately 240–310 ns in the system. The deviation plots and mean ± SEM of the receptor binding complex are shown in Table 3.

**FIGURE 14:**
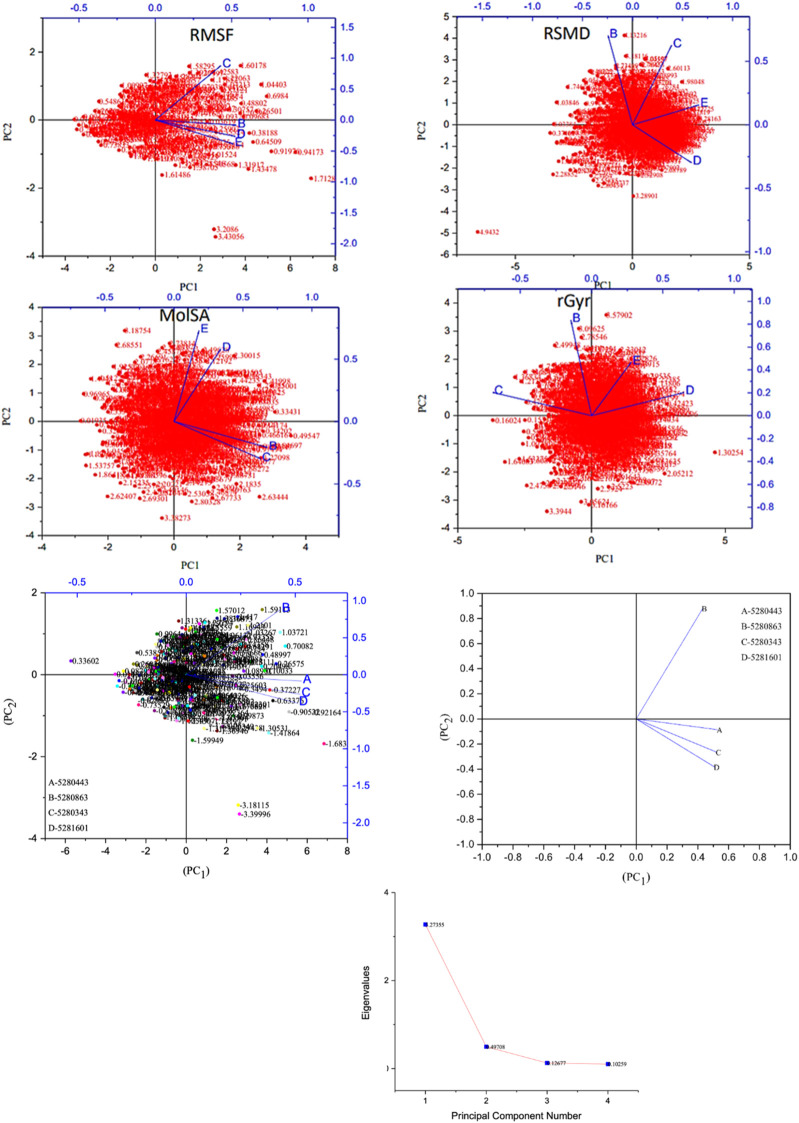
Principal component analysis of the native protein and ligands: (B) 5280443 (apigenin), (C) 5280863 (kaempferol), (D) 5280343 (quercetin), and (E) 5281601 (5-hydroxy-7,4-dimethoxyflavone).

**FIGURE 15:**
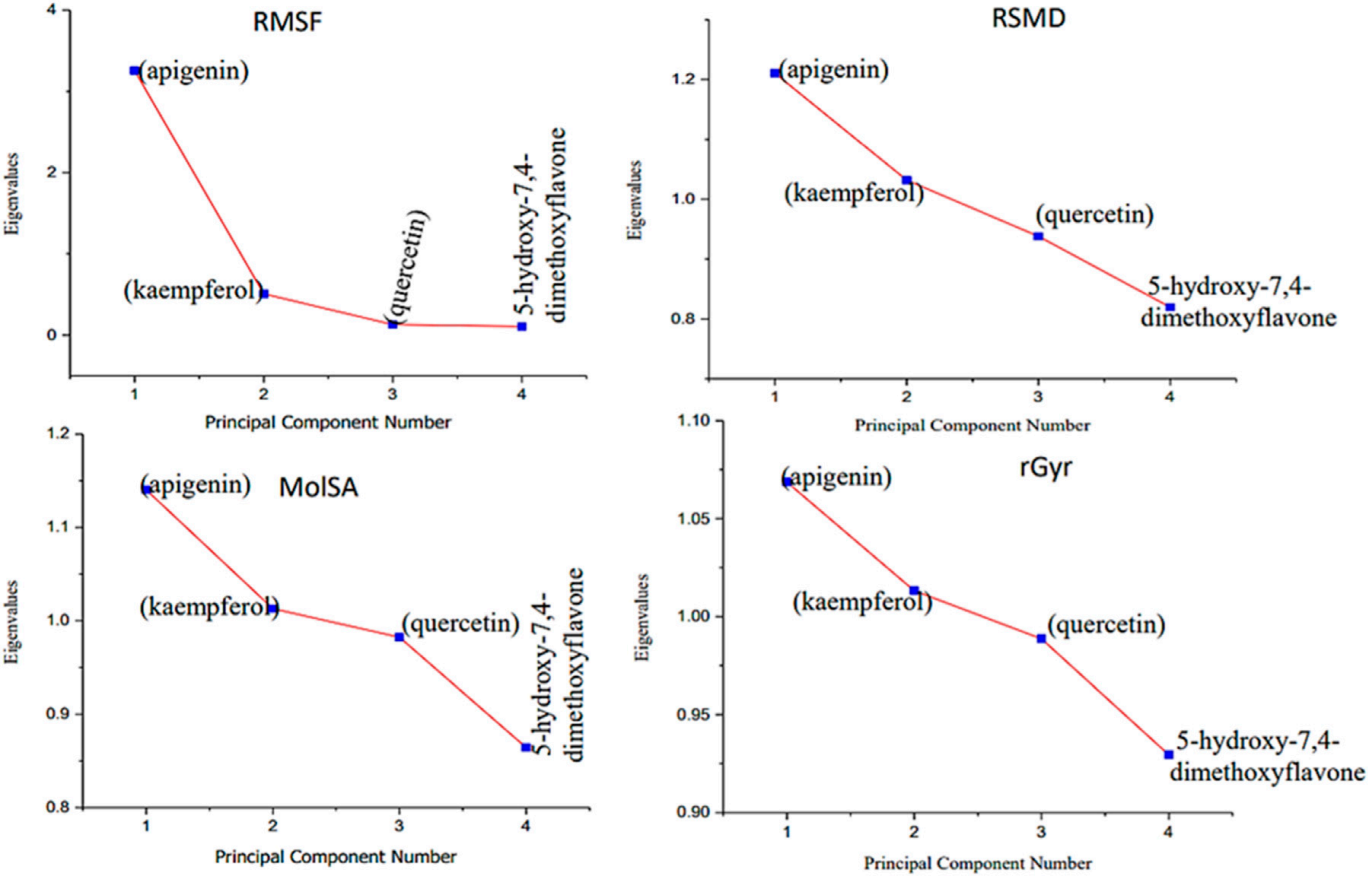
Eigenvalues against principal component analysis of the native protein and ligands: **(A)** 5280443 (apigenin), **(B)** 5280863 (kaempferol), **(C)** 5280343 (quercetin), and **(D)** 5281601 (5-hydroxy-7,4-dimethoxyflavone).

**FIGURE 16:**
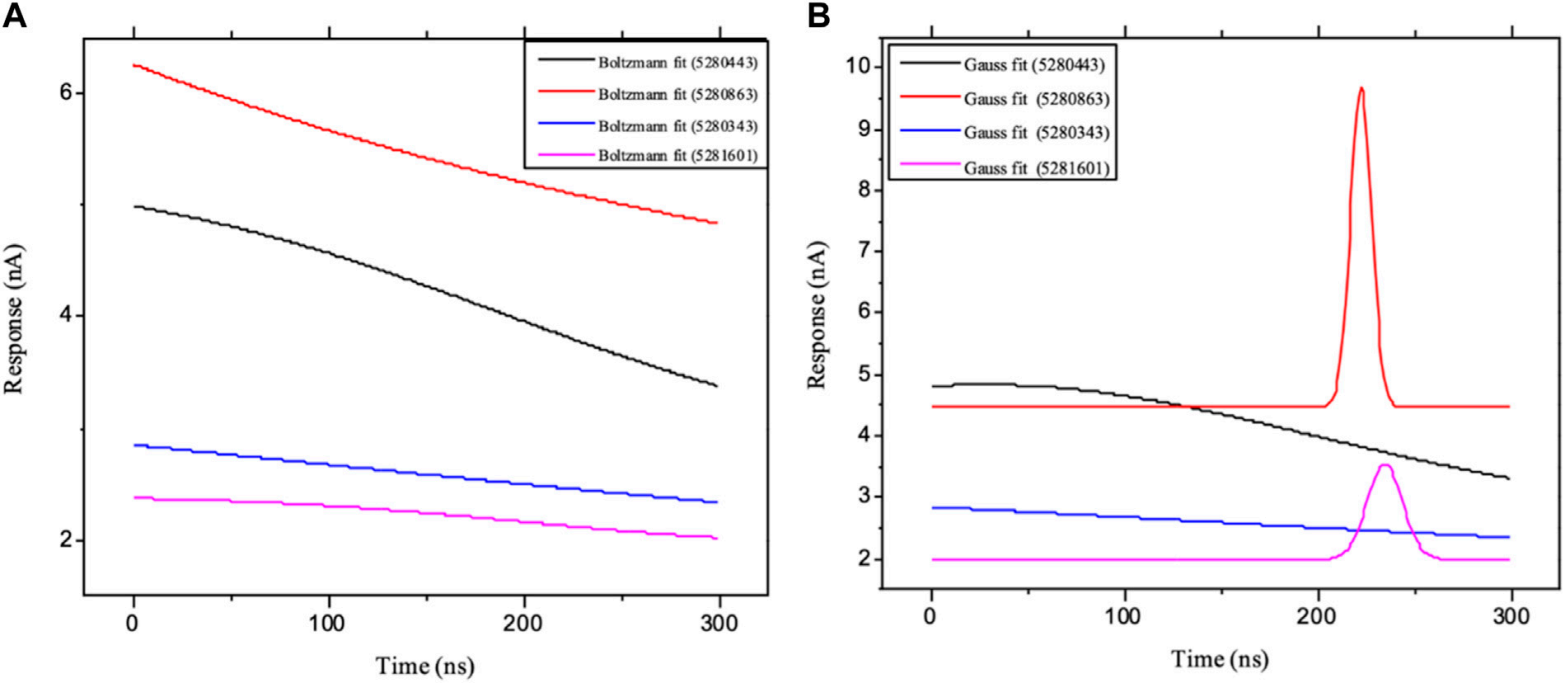
Schematic illustration of **(A)** Boltzmann and **(B)** Gaussian accelerated molecular dynamics of the ligands: ligands 5280343 (quercetin), 5280443 (apigenin), 5280863 (kaempferol) and 5281601 (5-hydroxy-7,4-dimethoxyflavone).

Moreover, the primary component, which examines the covariance matrix of the positional variances of the simulated receptors, is a key aspect studied to enhance the understanding of the structural stability of the residues towards a group of linearly related and unrelated variables. Consequently, as depicted in Figure 14, the scatter plot generated for the receptors and ligands clearly demonstrates significant differences in the ligands. Furthermore, through the diagonalization of the covariance matrix and utilization of the Origin-pro interface, as shown in Figure 15, the eigenvalues are revealed, providing essential information about the interconnected movements among the receptors. In summary, our results indicate that the ligands isolated from the fractionated extract of P. sarmentosum Roxb leaf display minor deviations in pattern and fluctuations compared to those observed in the reference. Furthermore, the dynamics of biomolecules often undergo high levels of structural changes during their biological functions, thereby resulting in distinct conformal states. In addition, both Gaussian and Boltzmann fitting accelerated molecular dynamics approaches can be used to simultaneously improve both the sampling and free energy calculations of these biomolecules. Therefore, our data, as shown in Figure 16, revealed that both Gaussian and Boltzmann fitting distributions were useful for further exploring the enhancement of sampling and free energy calculations of the biomolecules. However, the findings of the present study suggest that the selected ligands might be favorable for total cure of rheumatoid arthritis. Therefore, additional clinical studies are needed to determine the underlying mechanism involved and possibly to validate whether any of the selected ligands could be effective in the treatment of rheumatoid arthritis (Table 4).

**TABLE 4:** Principal component analysis of the native protein and ligands of interest.

Receptor (M)	Ligand	Eigen value cumulative	Percentage of variance	Coefficient of PC2	Coefficient of PC1
1P9M	5280443	3.273	81.84	−0.08588	0.529
	5280863	0.497	94.27	0.08826	0.431
	5280343	0.126	97.44	−0.26515	0.522
	5281601	0.102	100	−0.38402	0.510

5280343: Quercetin; 5280443: apigenin; 5280863: kaempferol; 5281601: 5-hydroxy-7, 4-dimethoxyflavone; PC: principal component analysis.

## 5 Conclusion

Our current study used a steadfast approach to identify potential immune protein inhibitors from a natural source (*Piper sarmentosum* Roxb leaf fractionated extract) as potential therapeutic agents for rheumatoid arthritis treatment. An *in silico* study conducted as part of this research has identified phyto-compounds 5280343 (quercetin), 5280443 (apigenin), 5280863 (kaempferol), and 5281601 (5-hydroxy-7,4-dimethoxyflavone) as promising inhibitors of these immune proteins. Furthermore, these phytocompounds that adhere to the Lipinski ROF and other significant parameters need to be considered drug candidates. However, several limitations and defects exist; for example, determining the accuracy and speed of docking calculations is challenging when exploring this approach could serve as a solution for most docking changes. Furthermore, the limited knowledge in academic computational models could be one of the challenges; however, further steps are needed to address these challenges via the integration of ligands and target of interest-, phenotype-, and biological network-based approaches with deeper reinforcement learning techniques, which could increase the predictive power of the models. However, *in vivo* and/or *in vitro* assays could further affirm the potency of these compounds as anti-inflammatory drugs for inhibiting IL-6 production.

## Data Availability

The datasets presented in this study can be found in online repositories. The names of the repository/repositories and accession number(s) can be found in the article/Supplementary Material.

## References

[B1] Adekiya T. A. Aruleba R. T. Klein A. Fadaka A. O. (2022). *In silico* inhibition of SGTP4 as a therapeutic target for the treatment of schistosomiasis. J. Biomol. Struct. Dyn. 40, 3697–3705. 10.1080/07391102.2020.1850363 33225839

[B2] Akmal M. N. Abdel Aziz I. Nur Azlina M. F. (2022). *Piper sarmentosum* Roxb. methanolic extract prevents stress-induced gastric ulcer by modulating oxidative stress and inflammation. Front. Pharmacol. 13, 971443. 10.3389/fphar.2022.971443 36712695 PMC9879357

[B3] Aletaha D. Smolen J. S. (2018). Diagnosis and management of rheumatoid arthritis: a review. JAMA 320, 1360–1372. 10.1001/jama.2018.13103 30285183

[B4] Alivernini S. Firestein G. S. McInnes I. B. (2022). The pathogenesis of rheumatoid arthritis. Immunity 55, 2255–2270. 10.1016/j.immuni.2022.11.009 36516818

[B5] Amaya-Amaya J. Botello-Corzo D. Calixto O. J. Calderón-Rojas R. Domínguez A. M. Cruz-Tapias P. (2012). Usefulness of patients-reported outcomes in rheumatoid arthritis focus group. Arthritis 2012, 935187. 10.1155/2012/935187 23097701 PMC3465872

[B6] Amaya-Amaya J. Sarmiento-Monroy J. C. Mantilla R. D. Pineda-Tamayo R. Rojas-Villarraga A. Anaya J. M. (2013). Novel risk factors for cardiovascular disease in rheumatoid arthritis. Immunol. Res. 56, 267–286. 10.1007/s12026-013-8398-7 23584985

[B7] Aryaeian N. Shahram F. Mahmoudi M. Tavakoli H. Yousefi B. Arablou T. (2019). The effect of ginger supplementation on some immunity and inflammation intermediate genes expression in patients with active Rheumatoid Arthritis. Gene 698, 179–185. 10.1016/j.gene.2019.01.048 30844477

[B8] Borhani D. W. Shaw D. E. (2012). The future of molecular dynamics simulations in drug discovery. J. Comput. Aided Mol. Des. 26, 15–26. 10.1007/s10822-011-9517-y 22183577 PMC3268975

[B9] Chaveerach A. Mokkamul P. Sudmoon R. Tanee T. (2006). Ethnobotany of the genus piper (Piperaceae) in Thailand.

[B10] Chi Vo V. (1997). Dictionary of medicinal plants in Vietnam. Ha Noi, Vietnam: Medical Publishing House.

[B11] Coleman R. A. (2011). Human tissue in the evaluation of safety and efficacy of new medicines: a viable alternative to animal models? ISRN Pharm. 2011, 806789. 10.5402/2011/806789 22389860 PMC3263708

[B12] Daily J. W. Yang M. Park S. (2016). Efficacy of turmeric extracts and curcumin for alleviating the symptoms of joint arthritis: a systematic review and meta-analysis of randomized clinical trials. J. Med. Food 19, 717–729. 10.1089/jmf.2016.3705 27533649 PMC5003001

[B13] Dayer J.-M. Choy E. (2010). Therapeutic targets in rheumatoid arthritis: the interleukin-6 receptor. Rheumatol. Oxf. 49, 15–24. 10.1093/rheumatology/kep329 PMC2789585 19854855

[B14] Do Tat L. (2004). Vietnamese medicinal plants and remedies. Hanoi, Vietnam: Medicine Publishing house.

[B15] Durrant J. D. McCammon J. A. (2011). Molecular dynamics simulations and drug discovery. BMC Biol. 9, 71. 10.1186/1741-7007-9-71 22035460 PMC3203851

[B16] Ekeuku S. O. Chin K.-Y. Mohd Ramli E. S. (2023). Effects of *Piper sarmentosum* on bone Health and fracture healing: a scoping review. Endocr. Metab. Immune Disord. Drug Targets 23, 908–916. 10.2174/1871530323666221130152737 36453484

[B17] Ekins S. Mestres J. Testa B. (2007). *In silico* pharmacology for drug discovery: methods for virtual ligand screening and profiling. Br. J. Pharmacol. 152, 9–20. 10.1038/sj.bjp.0707305 17549047 PMC1978274

[B18] Ernst E. Posadzki P. (2011). Complementary and alternative medicine for rheumatoid arthritis and osteoarthritis: an overview of systematic reviews. Curr. Pain Headache Rep. 15, 431–437. 10.1007/s11916-011-0227-x 21979101

[B19] Favalli E. G. (2020). Understanding the role of interleukin-6 (IL-6) in the joint and beyond: a comprehensive review of IL-6 inhibition for the management of rheumatoid arthritis. Rheumatol. Ther. 7, 473–516. 10.1007/s40744-020-00219-2 32734482 PMC7410942

[B20] Finckh A. Gilbert B. Hodkinson B. Bae S. C. Thomas R. Deane K. D. (2022). Global epidemiology of rheumatoid arthritis. Nat. Rev. Rheumatol. 18, 591–602. 10.1038/s41584-022-00827-y 36068354

[B21] Fraenkel L. Bathon J. M. England B. R. St Clair E. W. Arayssi T. Carandang K. (2021). 2021 American College of Rheumatology guideline for the treatment of rheumatoid arthritis. Arthritis Rheumatol. 73, 1108–1123. 10.1002/art.41752 34101376

[B22] Genheden S. Ryde U. (2015). The MM/PBSA and MM/GBSA methods to estimate ligand-binding affinities. Expert Opin. Drug Discov. 10, 449–461. 10.1517/17460441.2015.1032936 25835573 PMC4487606

[B23] Hematpoor A. Paydar M. Liew S. Y. Sivasothy Y. Mohebali N. Looi C. Y. (2018). Phenylpropanoids isolated from Piper sarmentosum Roxb. induce apoptosis in breast cancer cells through reactive oxygen species and mitochondrial-dependent pathways. Chem. Biol. Interact. 279, 210–218. 10.1016/j.cbi.2017.11.014 29174417

[B24] Hirano T. (2021). IL-6 in inflammation, autoimmunity and cancer. Int. Immunol. 33, 127–148. 10.1093/intimm/dxaa078 33337480 PMC7799025

[B25] Huang M.-C. Pai F. T. Lin C. C. Chang C. M. Chang H. H. Lee Y. C. (2015). Characteristics of traditional Chinese medicine use in patients with rheumatoid arthritis in Taiwan: a nationwide population-based study. J. Ethnopharmacol. 176, 9–16. 10.1016/j.jep.2015.10.024 26481605

[B26] Hussain K. Hashmi F. K. Latif A. Ismail Z. Sadikun A. (2012). A review of the literature and latest advances in research of Piper sarmentosum. Pharm. Biol. 50, 1045–1052. 10.3109/13880209.2011.654229 22486533

[B27] Li X.-Z. Zhang S.-N. (2020). Herbal compounds for rheumatoid arthritis: literature review and cheminformatics prediction. Phytother. Res. 34, 51–66. 10.1002/ptr.6509 31515874

[B28] Makchuchit S. Rattarom R. Itharat A. (2017). The anti-allergic and anti-inflammatory effects of Benjakul extract (a Thai traditional medicine), its constituent plants and its some pure constituents using *in vitro* experiments. Biomed. Pharmacother. 89, 1018–1026. 10.1016/j.biopha.2017.02.066 28292010

[B29] Masuda T. Inazumi A. Yamada Y. Padolina W. G. Kikuzaki H. Nakatani N. (1991). Antimicrobial phenylpropanoids from *Piper sarmentosum* . Phytochemistry 30, 3227–3228. 10.1016/0031-9422(91)83180-s

[B30] McInnes I. B. Schett G. (2007). Cytokines in the pathogenesis of rheumatoid arthritis. Nat. Rev. Immunol. 7, 429–442. 10.1038/nri2094 17525752

[B31] McInnes I. B. Schett G. (2011). The pathogenesis of rheumatoid arthritis. N. Engl. J. Med. 365, 2205–2219. 10.1056/NEJMra1004965 22150039

[B32] Miean K. H. Mohamed S. (2001). Flavonoid (myricetin, quercetin, kaempferol, luteolin, and apigenin) content of edible tropical plants. J. Agric. Food Chem. 49, 3106–3112. 10.1021/jf000892m 11410016

[B33] Mohd Zainudin M. Zakaria Z. Megat Mohd Nordin N. A. (2015). The use of *Piper sarmentosum* leaves aqueous extract (Kadukmy^TM^) as antihypertensive agent in spontaneous hypertensive rats. BMC Complement. Altern. Med. 15, 54. 10.1186/s12906-015-0565-z 25887182 PMC4367816

[B34] Najib Nik A Rahman N. Furuta T. kojima S. Takane K. Ali Mohd M. (1999). Antimalarial activity of extracts of Malaysian medicinal plants. J. Ethnopharmacol. 64, 249–254. 10.1016/s0378-8741(98)00135-4 10363840

[B35] Nguyen P. H. Tran V. D. Pham D. T. Dao T. N. P. Dewey R. S. (2021). Use of and attitudes towards herbal medicine during the COVID-19 pandemic: a cross-sectional study in Vietnam. Eur. J. Integr. Med. 44, 101328. 10.1016/j.eujim.2021.101328 36570027 PMC9760728

[B36] Nguyen T. K. Thuy Thi Tran L. Ho Viet D. Thai P. H. Ha T. P. Ty P. V. (2023). Xanthine oxidase, α-glucosidase and α-amylase inhibitory activities of the essential oil from Piper lolot: *in vitro* and *in silico* studies. Heliyon 9, e19148. 10.1016/j.heliyon.2023.e19148 37636421 PMC10458695

[B37] Ojo O. A. Adegboyega A. E. Johnson G. I. Umedum N. L. Onuh K. Adeduro M. N. (2021). Deciphering the interactions of compounds from Allium sativum targeted towards identification of novel PTP 1B inhibitors in diabetes treatment: a computational approach. Inf. Med. Unlocked 26, 100719. 10.1016/j.imu.2021.100719

[B38] Onikanni A. S. Lawal B. Oyinloye B. E. Mostafa-Hedeab G. Alorabi M. Cavalu S. (2022). Therapeutic efficacy of Clompanus pubescens leaves fractions via downregulation of neuronal cholinesterases/Na+-K+ATPase/IL-1 β, and improving the neurocognitive and antioxidants status of streptozotocin-induced diabetic rats. Biomed. Pharmacother. 148, 112730. 10.1016/j.biopha.2022.112730 35183996

[B39] Othman N. S. Che Roos N. A. Aminuddin A. Murthy J. K. A Hamid A. Ugusman A. (2022). Effects of *Piper sarmentosum* Roxb. on hypertension and diabetes mellitus: a systematic review and meta-analysis. Front. Pharmacol. 13, 976247. 10.3389/fphar.2022.976247 36091787 PMC9453491

[B40] Purba R. A. P. Paengkoum S. Paengkoum P. (2021). Development of a simple high-performance liquid chromatography-based method to quantify synergistic compounds and their composition in dried leaf extracts of *piper sarmentosum* Roxb. Separations 8, 152. 10.3390/separations8090152

[B41] Ridtitid W. Rattanaprom W. Thaina P. Chittrakarn S. Sunbhanich M. (1998). Neuromuscular blocking activity of methanolic extract of *Piper sarmentosum* leaves in the rat phrenic nerve-hemidiaphragm preparation. J. Ethnopharmacol. 61, 135–142. 10.1016/s0378-8741(98)00025-7 9683344

[B42] Scott D. L. Wolfe F. Huizinga T. W. (2010). Rheumatoid arthritis. Lancet 376, 1094–1108. 10.1016/S0140-6736(10)60826-4 20870100

[B43] Sim K.-M. Mak C.-N. Ho L.-P. (2009). A new amide alkaloid from the leaves of *Piper sarmentosum* . J. Asian Nat. Prod. Res. 11, 757–760. 10.1080/10286020903058933 20183320

[B44] Soeken K. L. Miller S. A. Ernst E. (2003). Herbal medicines for the treatment of rheumatoid arthritis: a systematic review. Rheumatol. Oxf. 42, 652–659. 10.1093/rheumatology/keg183 12709541

[B45] Srirangan S. Choy E. H. (2010). The role of interleukin 6 in the pathophysiology of rheumatoid arthritis. Ther. Adv. Musculoskelet. Dis. 2, 247–256. 10.1177/1759720X10378372 22870451 PMC3383508

[B46] Suhana Mohd Ramli E. Nirwana Soelaiman I. Othman F. Ahmad F. Nazrun Shuib A. Mohamed N. (2012). The effects of *piper sarmentosum* water extract on the expression and activity of 11β-hydroxysteroid dehydrogenase type 1 in the bones with excessive glucocorticoids. Iran. J. Med. Sci. 37, 39–46. 23115429 PMC3470290

[B47] Sun X. Chen W. Dai W. Xin H. Rahmand K. Wang Y. (2020). *Piper sarmentosum* Roxb. a review on its botany, traditional uses, phytochemistry, and pharmacological activities. J. Ethnopharmacol. 263, 112897. 10.1016/j.jep.2020.112897 32620264

[B48] Takeuchi T. Yoshida H. Tanaka S. (2021). Role of interleukin-6 in bone destruction and bone repair in rheumatoid arthritis. Autoimmun. Rev. 20, 102884. 10.1016/j.autrev.2021.102884 34229044

[B49] Udenze E. C. C. Ezirim A. Ihedimbu C. P. Iheme C. (2014). Effects of oral administration of Garcinia kola seeds on haematological and defence parameters of diabetic rats. Am. J. Biochem. Mol. Biol. 4, 167–175. 10.3923/ajbmb.2014.167.175

[B50] Ugusman A. Zakaria Z. Hui C. K. Nordin N. A. M. M. (2011). *Piper sarmentosum* inhibits ICAM-1 and Nox4 gene expression in oxidative stress-induced human umbilical vein endothelial cells. BMC Complement. Altern. Med. 11, 31. 10.1186/1472-6882-11-31 21496279 PMC3090383

[B51] Ugusman A. Zakaria Z. Hui C. K. Nordin N. A. M. M. Mahdy Z. A. (2012). Flavonoids of *Piper sarmentosum* and its cytoprotective effects against oxidative stress. EXCLI J. 11, 705–714. 27847456 PMC5099915

[B52] Umar H. I. Josiah S. S. Saliu T. P. Jimoh T. O. Ajayi A. Danjuma J. B. (2021). In-silico analysis of the inhibition of the SARS-CoV-2 main protease by some active compounds from selected African plants. J. Taibah Univ. Med. Sci. 16, 162–176. 10.1016/j.jtumed.2020.12.005 33437230 PMC7787523

[B53] Vimala S. Mohd I. A. Abdull R. A. Rohana S. (2003). Natural antioxidants: *Piper sarmentosum* (kadok) and Morinda elliptica (mengkudu). Malays. J. Nutr. 9, 41–51. 22692531

[B54] Wang D. F. Zhou L. L. Zhou H. L. Hou G. Y. Zhou X. Li W. (2017). Effects of *Piper sarmentosum* extract on the growth performance, antioxidant capability and immune response in weaned piglets. J. Anim. Physiol. Anim. Nutr. Berl. 101, 105–112. 10.1111/jpn.12517 27045971

[B55] Ware I. Franke K. Dube M. Ali El Enshasy H. Wessjohann L. A. (2023). Characterization and bioactive potential of secondary metabolites isolated from *Piper sarmentosum* Roxb. Int. J. Mol. Sci. 24, 1328. 10.3390/ijms24021328 36674844 PMC9862425

[B56] Xu X. Xu H. Shang Y. Zhu R. Hong X. Song Z. (2021). Development of the general chapters of the Chinese Pharmacopoeia 2020 edition: a review. J. Pharm. Anal. 11, 398–404. 10.1016/j.jpha.2021.05.001 34513116 PMC8424356

[B57] Yeo E. T. Y. Wong K. W. L. See M. L. Wong K. Y. Gan S. Y. Chan E. W. L. (2018). Piper sarmentosum Roxb. confers neuroprotection on beta-amyloid (Aβ)-induced microglia-mediated neuroinflammation and attenuates tau hyperphosphorylation in SH-SY5Y cells. J. Ethnopharmacol. 217, 187–194. 10.1016/j.jep.2018.02.025 29462698

[B58] Yeoh H.-H. Wong P.-F. M. (1993). Food value of lesser utilised tropical plants. Food Chem. 46, 239–241. 10.1016/0308-8146(93)90113-t

[B59] Yoshida Y. Tanaka T. (2014). Interleukin 6 and rheumatoid arthritis. Biomed. Res. Int. 2014, 698313. 10.1155/2014/698313 24524085 PMC3913495

[B60] Zainab B. Ayaz Z. Alwahibi M. S. Khan S. Rizwana H. Soliman D. W. (2020). In-silico elucidation of Moringa oleifera phytochemicals against diabetes mellitus. Saudi J. Biol. Sci. 27, 2299–2307. 10.1016/j.sjbs.2020.04.002 32884411 PMC7451590

[B61] Zainal Ariffin S. H. Wan Omar W. H. H. Zainal Ariffin Z. Safian M. F. Senafi S. Megat Abdul Wahab R. (2009). Intrinsic anticarcinogenic effects of *Piper sarmentosum* ethanolic extract on a human hepatoma cell line. Cancer Cell Int. 9, 6. 10.1186/1475-2867-9-6 19257877 PMC2667431

[B62] Zainol Abidin I. Z. Johari A. N. Yazid M. D. Zainal Ariffin Z. Eziwar Dyari H. R. Zainal Ariffin S. H. (2023). Osteogenic potential and bioactive profiles of *Piper sarmentosum* ethanolic extract-treated stem cells. Pharmaceuticals 16, 708. 10.3390/ph16050708 37242491 PMC10222704

[B63] Zakaria Z. A. Patahuddin H. Mohamad A. S. Israf D. A. Sulaiman M. R. (2010). *In vivo* anti-nociceptive and anti-inflammatory activities of the aqueous extract of the leaves of Piper sarmentosum. J. Ethnopharmacol. 128, 42–48. 10.1016/j.jep.2009.12.021 20035852

[B64] Zeng L. Yang T. Yang K. Yu G. Li J. Xiang W. (2022). Efficacy and safety of curcumin and curcuma longa extract in the treatment of arthritis: a systematic review and meta-analysis of randomized controlled trial. Front. Immunol. 13, 891822. 10.3389/fimmu.2022.891822 35935936 PMC9353077

[B65] Zhang Y. Mao X. Li W. Chen W. Wang X. Ma Z. (2021). Tripterygium wilfordii: an inspiring resource for rheumatoid arthritis treatment. Med. Res. Rev. 41, 1337–1374. 10.1002/med.21762 33296090

