# Supplementary material for: In silico identification of compounds from Piper sarmentosum Roxb leaf fractionated extract inhibit interleukin-6 to prevent rheumatoid arthritis
Source: Front Pharmacol. 2024 Mar 21;15:1358037. doi: 10.3389/fphar.2024.1358037 (PMC10991700; doi:10.3389/fphar.2024.1358037)
Supplement: Supplementary file 2 [file Table1.DOCX]

**Supporting Information:**

**Selected compounds from** ***Piper sarmentosum Roxb leaf* fractionated extract inhibit Interleukin-6 (IL-6) to prevent Rheumatoid Arthritis –Computational Prognosis**

**Tran Nhat Phong Dao^1, 2^, Sunday Amos Onikanni ^3, 4^*****, Adewale Oluwaseun Fadaka^5^, Ashwil Klein^5^, Van De Tran^7^, Minh Hoang Le^2^, Hen-Hong Chang^1, 7 , 8^***

^1^Graduate Institute of Integrated Medicine, College of Chinese Medicine, China Medical University, Taichung 40402, Taiwan (ROC)

^2^Department of Traditional Medicine, Can Tho University of Medicine and Pharmacy, Can Tho 900000, Vietnam

^3^College of Medicine, Graduate Institute of Biomedical Sciences, China Medical University, Taiwan (ROC)

^4^Department of Chemical Sciences, Biochemistry Unit, Afe-Babalola University, Ado-Ekiti, Ekiti State, Nigeria

^5^Department of Biotechnology, University of the Western Cape, Bellville, South Africa

^6^ Department of Health Organization and Management, Can Tho University of Medicine and Pharmacy, Can Tho 900000, Vietnam

^7^ Chinese Medicine Research Center, China Medical University, Taichung 40402, Taiwan (ROC)

^8^ Department of Chinese Medicine, China Medical University Hospital, Taichung 40402, Taiwan (ROC)

*Authors to whom correspondence should be addressed:u109305123@cmu.edu.tw (S.A.O.) and tcmchh55@mail.cmu.edu.tw (H.H.C)

**Data curation and software availability**

The Protein Data Bank (PDB:1P9M) repository (<https://www.rcsb.org/>) was used to retrieve three dimensional (3D) X-ray crystal structure of Interleukin-6 (IL-6) receptor, followed by the removal of the co-crystallized ligands. Next, glide's protein preparation wizard panel (Schrödinger Suite 2022-3) (<https://pubchem.ncbi.nlm.nih.gov/>).

**Ligand preparation**

The LigPrep module from Schrödinger Suite 2022-3 was accustomed to prepare a total of fifty-five (55) phytocompounds of *Piper sarmentosum Roxb* leaf fractionated extract identified from ethnobotanical databases for molecular docking^[S1,2]^. The 3D structures were created on low energy with the proper chiralities. At a physiological pH of 7.2± 0.2, the possible ionization states for every ligand structure were generated. Each ligand's stereoisomers were calculated by keeping certain chiralities constant while varying other^S3^.

**Receptor grid generation**

For ligand docking, receptor grid generation allows determining the position and size of the protein's active region. Using the receptor grid construction tool in Schrödinger Maestro 12.5, the scoring grid was supported by the crystal structure of Interleukin-6 (IL-6). Nonpolar receptor atoms' van der Waals (vdW) radius scaling factor was set to 1.0, with a partial charge cut-off of 0.25. Consequently, the top-scoring compound from the molecular docking analysis was further subjected to induced fit docking using Maestro 12.5's induced fit docking panel.

**Protein-Ligand Docking**

The outcome of receptor grid file was utilized to perform molecular docking investigations with the Glide tool of Schrödinger Maestro 12.5. Standard precision (SP) was employed to dock the protein Interleukin-6 (IL-6) and therefore prepared ligands from *P. sarmentosum Roxb* along with the quality activator keeping the ligand sampling set to flexible and ligand sampling set to none (refine only). For ligand atoms, the vdW radius scaling factor was scaled at 0.80 with a partial charge cut-off of 0.15.

**Multiple-Ligand and Receptor-Ligand Complex Modelling**

The chemistry of the ligands was properly standardized and extrapolated, and the structure file (SDF) of the test compounds sourced from the PubChem database was prepared using the LigPrep panel of the Schrödinger suite (Schrödinger 2022-3, LLC, New York, NY, USA), and were used for modelling using PHASE. The PHASE aligned the ligands automatically and supported their optimum arrangement and mutual characteristics. PHASE was accustomed to create a receptor-ligand complex model employing the highest nine compounds with the very best binding affinity against the target protein in regard to the quality. The hypothesis was set with a maximum number of features to be created as 7.00, a minimum feature-feature distance of 2.00, a minimum feature-feature distance of 4.00 for components of the identical type, and donors as vectors. Aside from the donor and negative ionic features, which were set to 1, the hypothesis difference criteria were retained, and the acceptor and negative features were made equivalent.

**Binding Free Energy Calculation**

The Prime Molecular Mechanics-Generalized Born area MM-GBSA tool (Schrödinger suite 2022–3) was accustomed to determine the steadiness of protein-ligand complexes according to their binding free energy. The ligands were prepared beforehand using LigPrep, and therefore the relevant proteins were prepared using the protein preparation wizard, as detailed previously. Sitemap anticipated the active sites of the proteins. Glide standard precision (SP) docking was then accustomed to dock the chemicals with proteins. The MM-GBSA technology offered with Prime was utilized to work out binding free energy for ligand-protein complexes utilizing the Prime MM-GBSA panel. The OPLS3 physical phenomenon was chosen, and therefore the continuum solvent model was VSGB. The default settings for the opposite options were selected^S3^. Each of the ligand containing ΔGbind with LASV nucleoprotein were calculated on the basis of the following equation. ΔGbind = ΔE + ΔGsolv + ΔGSA (1) ΔE = E_complex_ - E_protein_ – E_ligand_ where, E_complex_, Eprotein and E_ligand_ are the minimized energies of the protein–inhibitor complex, protein, and inhibitor, respectively. Furthermore, ΔGsolv = Gsolv(_complex_) - Gsolv(protein) - Gsolv(_ligand_) where, Gsolv(_complex_), Gsolv(_protein_), and Gsolv(_ligand_) contains the salvation free energies of the complex, protein, and inhibitor, respectively. ΔGSA = GSA (_complex_) - GSA (_protein_) - GSA (_ligand_) where GSA (_complex_), GSA (_protein_), and GSA (_ligand_) served as surface area energies for the complex, protein, and inhibitor, respectively^S3^.

**Pharmacological Parameters**

The features of the test drugs' absorption, distribution, metabolism, excretion, and toxicity (ADMET) were assessed using in silico integrative model predictions at the SwissADME and PROTOX-II servers, respectively.

**Molecular Dynamics Simulations and Trajectory point Analysis**

Analysis of MD simulation for the receptor of interest (1P9M) was prepared using Schrödinger Suite 21.3 with Maestro version 12.5.137, MM share version 5.7.137, and Windows-x64 Platform. The buildup system of MD preparation and trajectory analysis methods were followed from previous similar research^S4^. Each of the docked complexes were individually subjected to Molecular Simulation following the Desmond module of the Schrödinger software with an OPLS 2005 force field. The protein–ligand complex was bounded with a predefined transferable intermolecular potential with a 3-point water model in an orthorhombic box. Neutralization of the overall charge was minimized with addition of sodium and chloride ions to mimic physiological conditions. Both temperature and pressure were kept constant at 310^0^C and 1.01325 bar respectively, by using a Nose-Hoover thermostat and a Martyna-Tobias-Klein barostat made from United State. The simulation relaxation was undertaken by using an NPT ensemble after considering the number of atoms, the pressure, and the timescale. During the MD simulation, the long-range electrostatic interactions were calculated by using the particle mesh Ewald method. Furthermore, analysis of MD simulation was carried out for 100 ns, and the trajectory sampling was set at an interval of 100 ps with 1000 frame numbers. Simulation outputs were analyzed and visualized by a simulation interaction diagram and an MS-MD trajectory analysis. The MD analysis was done in replicate to avoid variation. Data were plotted by using OriginPro version 9.


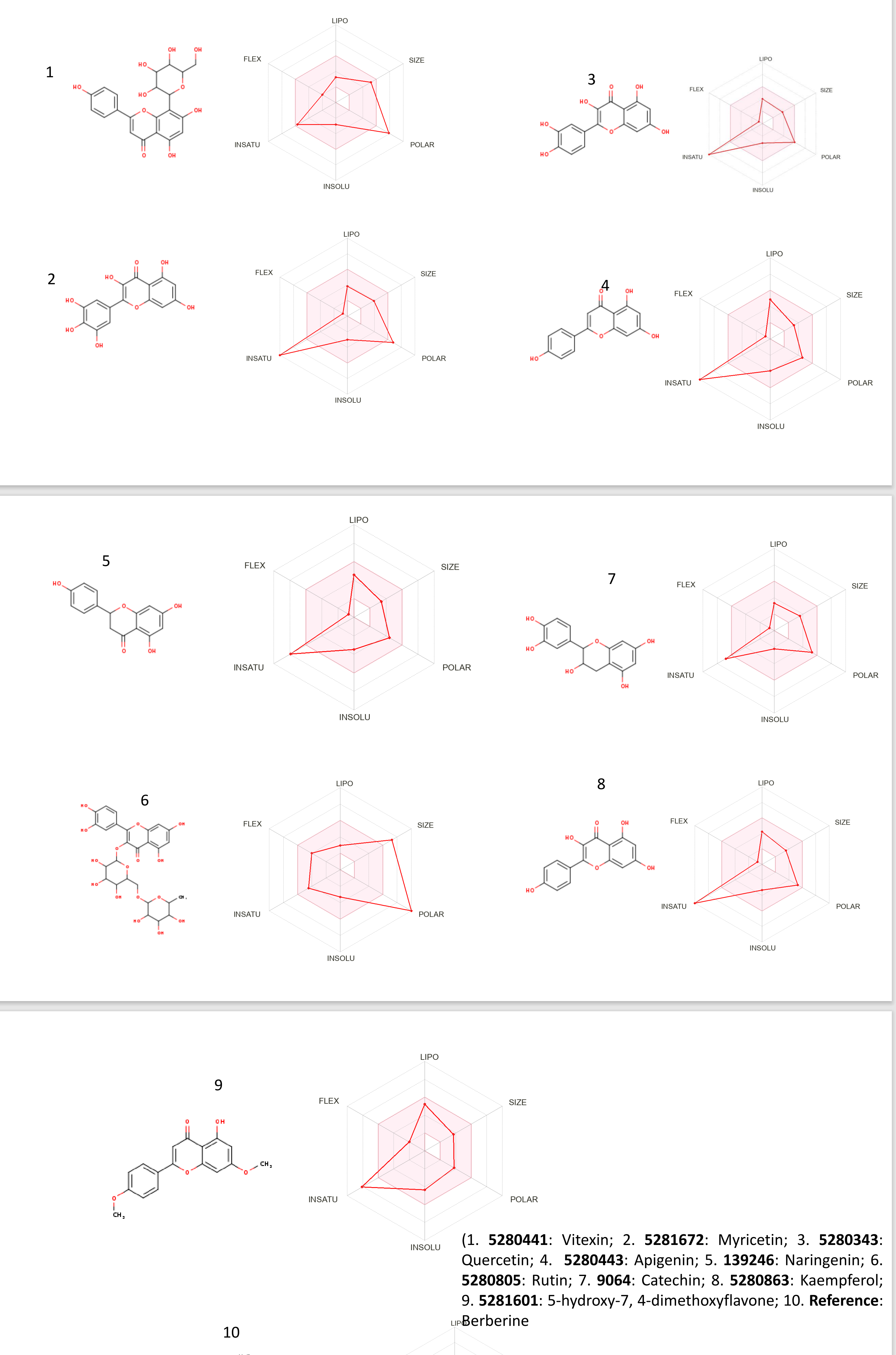


S1. Molecules obtained after using PubChem is highlighted.


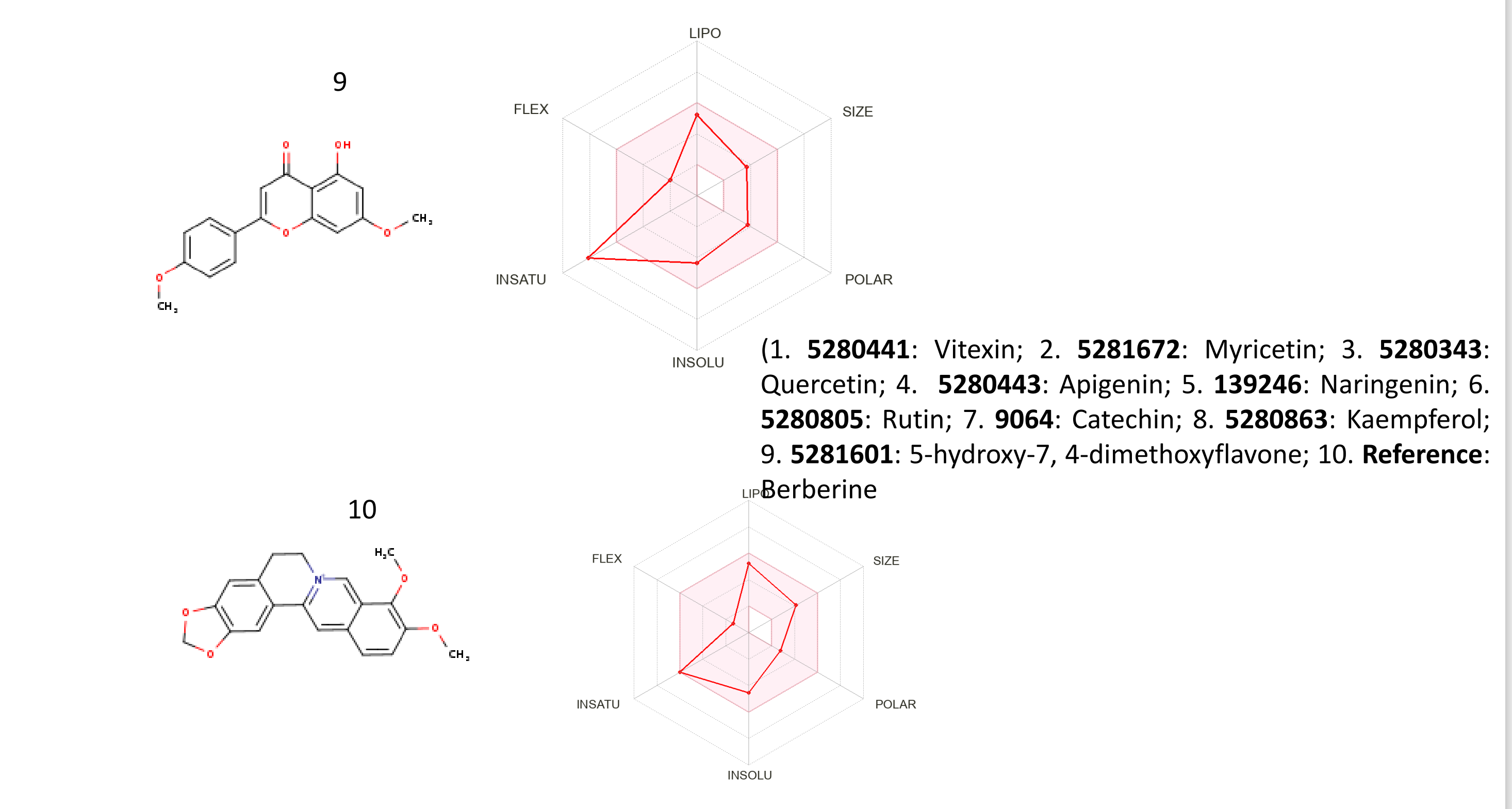


S2. Molecules obtained after using PubChem is highlighted.


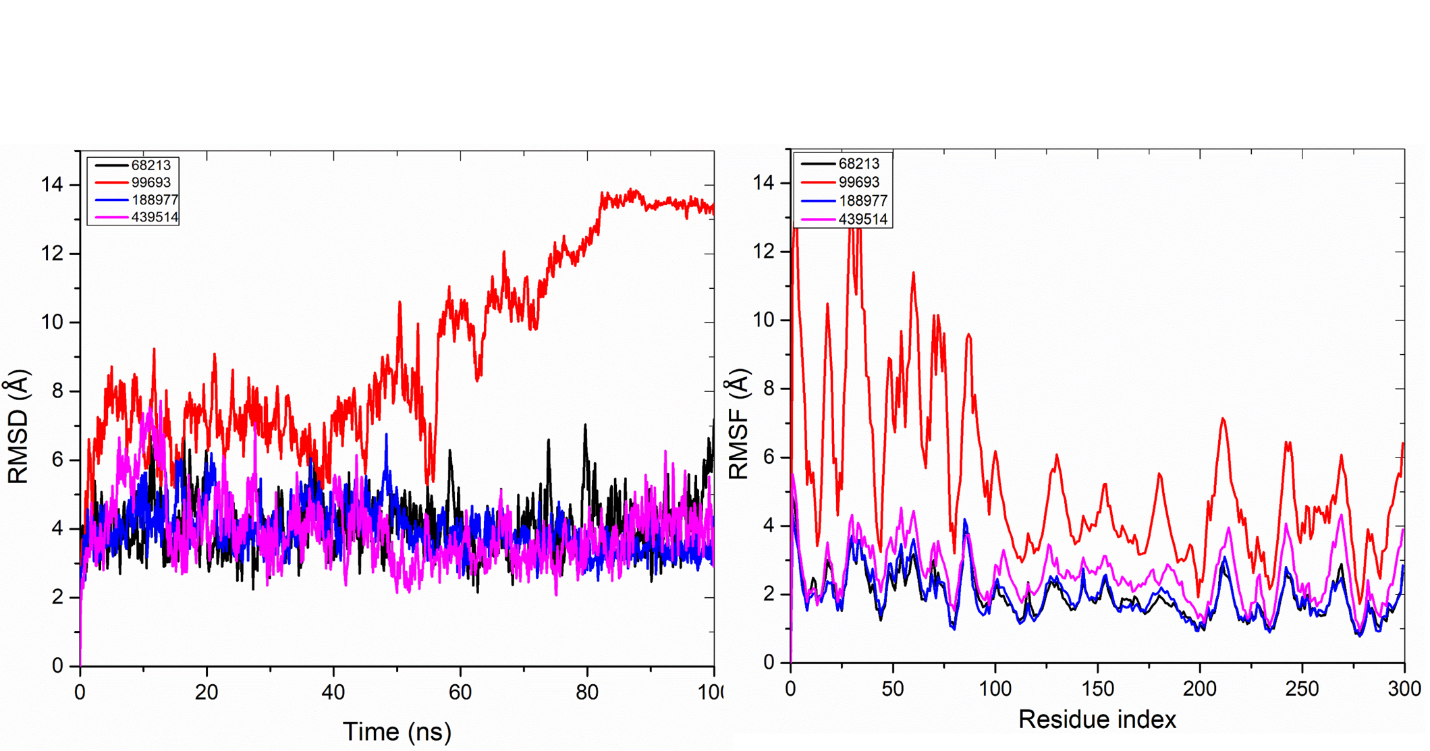


S3. MD simulation of 1P9M complexed to **5280343 (Quercetin); 5280443 (Apigenin); 5280863 (Kaempferol) and 5281601 (5-hydroxy-7, 4-dimethoxyflavone).** (A) P_RMSF and L_RMSD graphical illustration plot. All simulations were carried out using (Schrödinger suite version maestro v21.3).


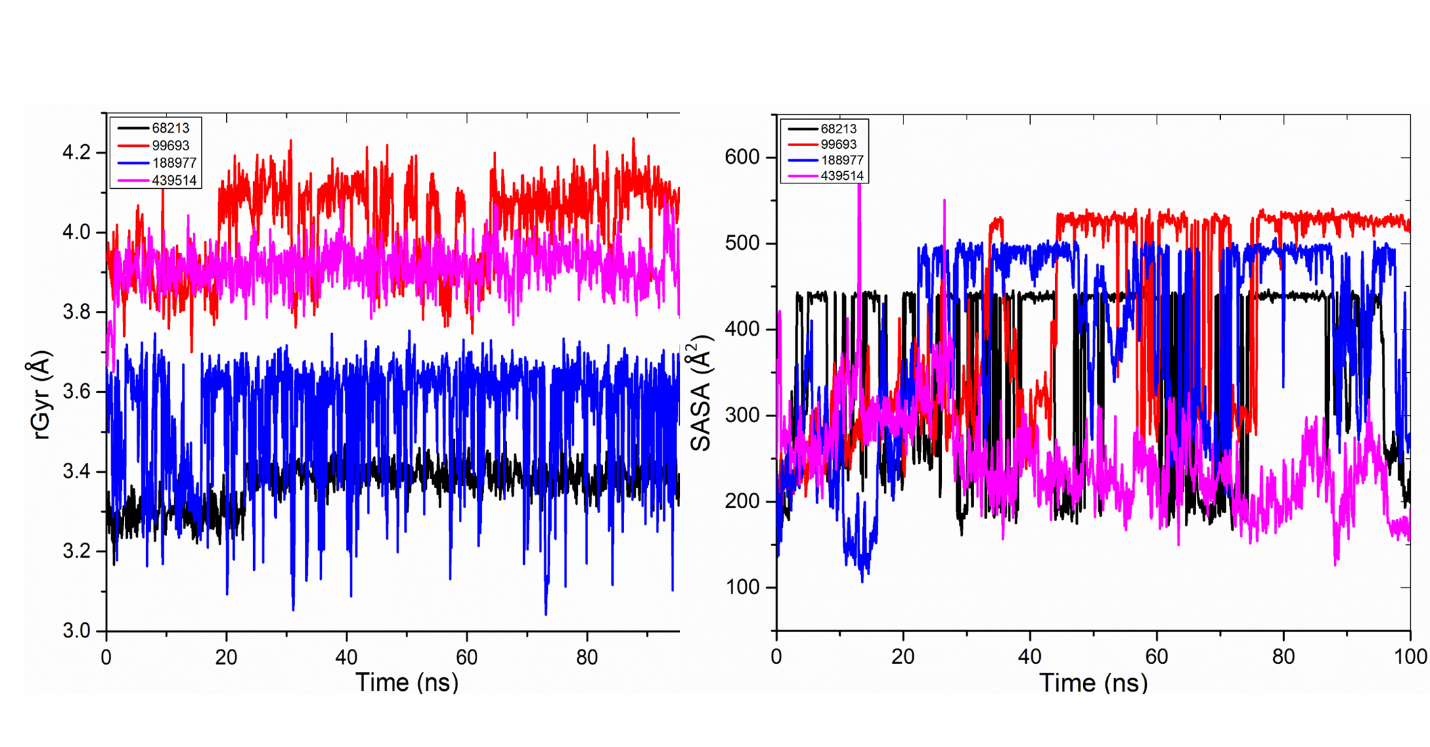


S4. MD simulation of 1P9M complexed to **5280343 (Quercetin); 5280443 (Apigenin); 5280863 (Kaempferol) and 5281601 (5-hydroxy-7, 4-dimethoxyflavone).** (A) MolSA representation, and (B) SASA diagram. All simulations were carried out using (Schrödinger suite version maestro v21.3).


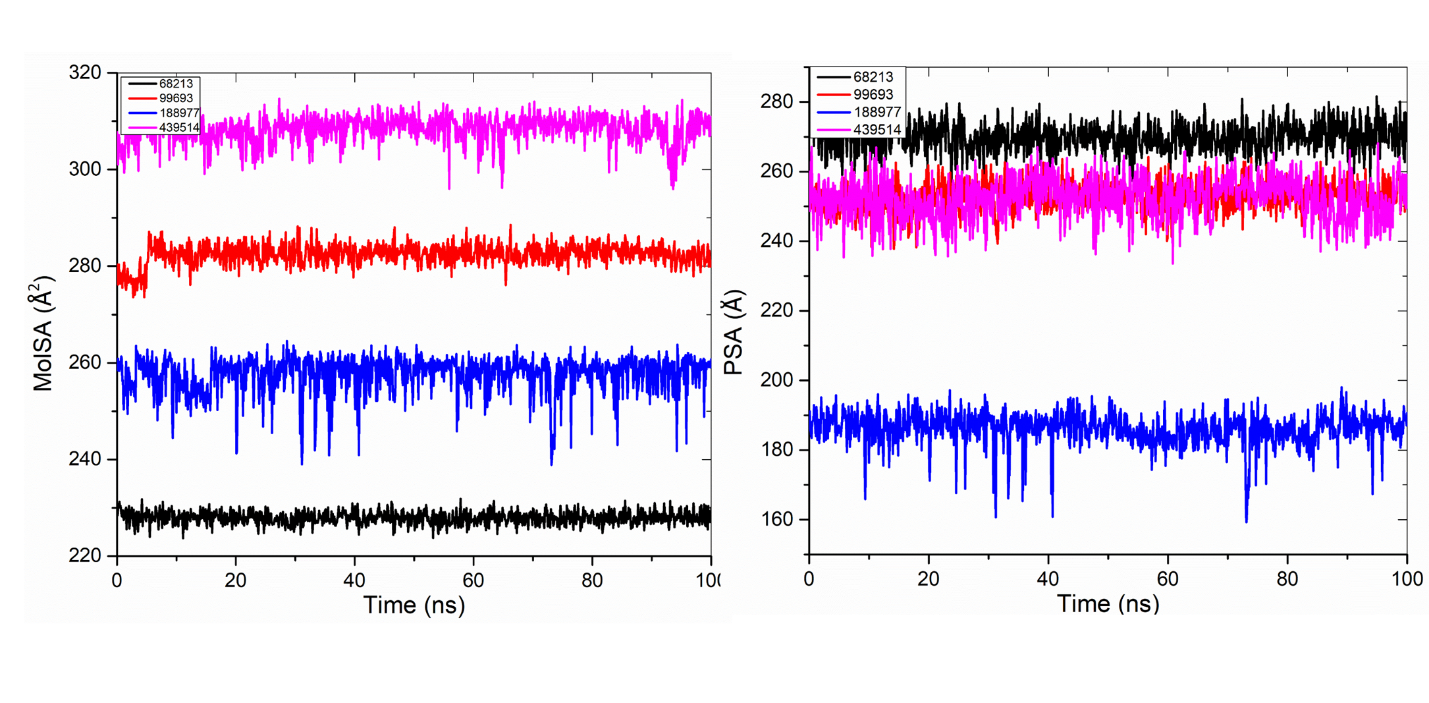


S5. MD simulation of 1P9M complexed to **5280343 (Quercetin); 5280443 (Apigenin); 5280863 (Kaempferol) and 5281601 (5-hydroxy-7, 4-dimethoxyflavone)**  (A) rGyr representation, and (B) PSA diagram. All simulations were carried out using (Schrödinger suite version maestro v21.3).


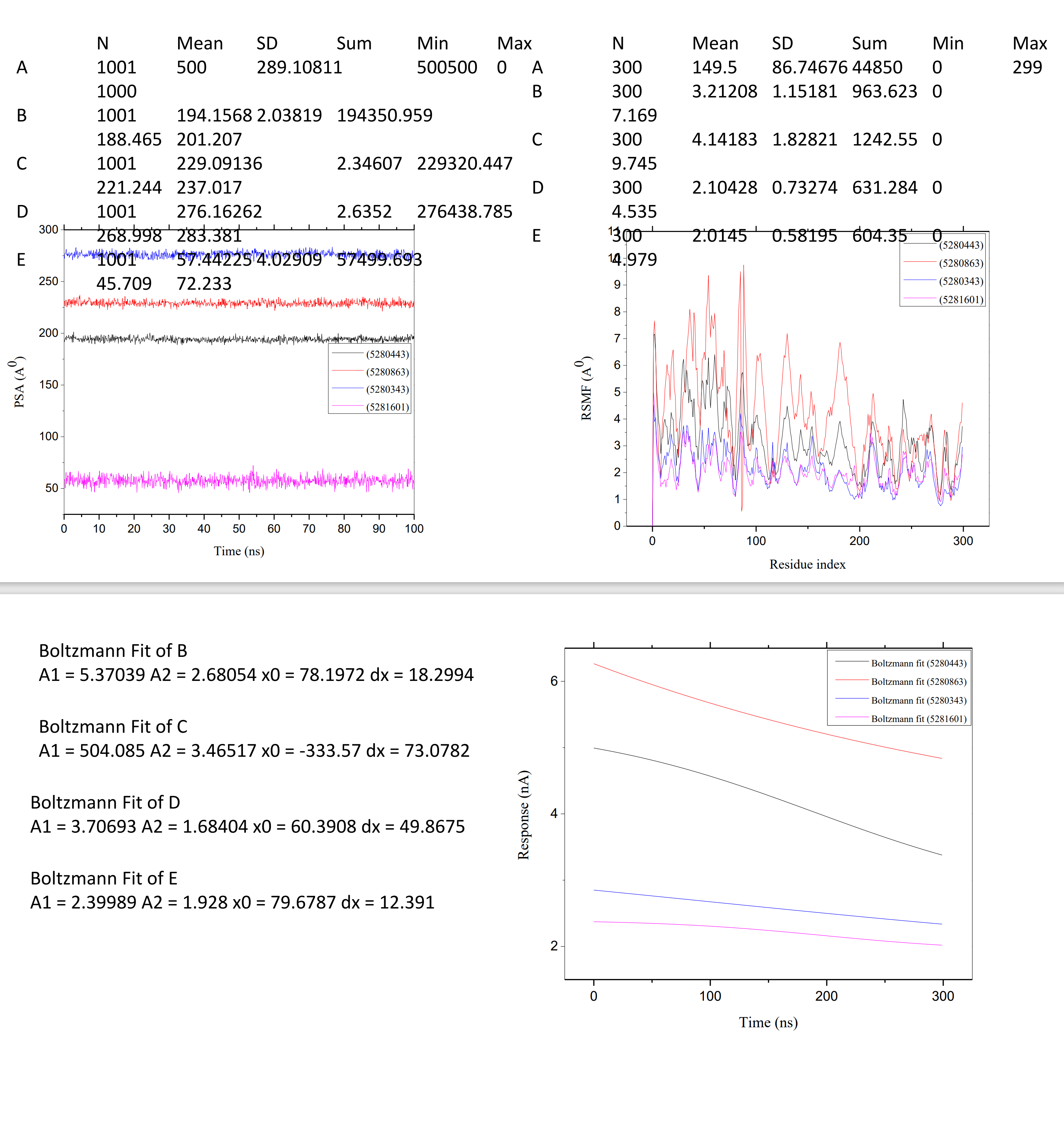


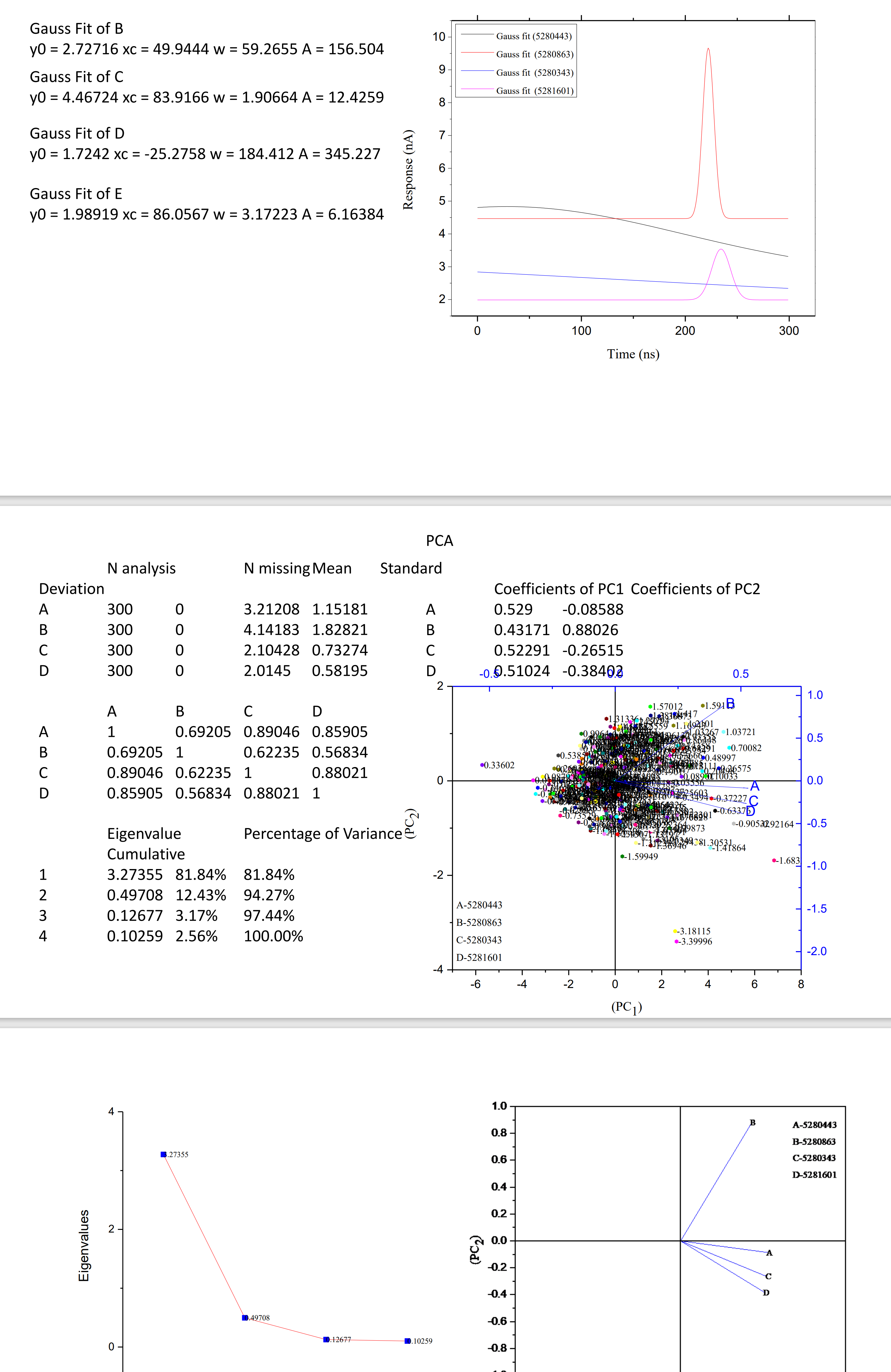


S6. Schematic illustration of Boltzmann and Gaussian accelerated molecular dynamics of the ligands: ligands: **5280343 (Quercetin); 5280443 (Apigenin); 5280863 (Kaempferol) and 5281601 (5-hydroxy-7, 4-dimethoxyflavone).**


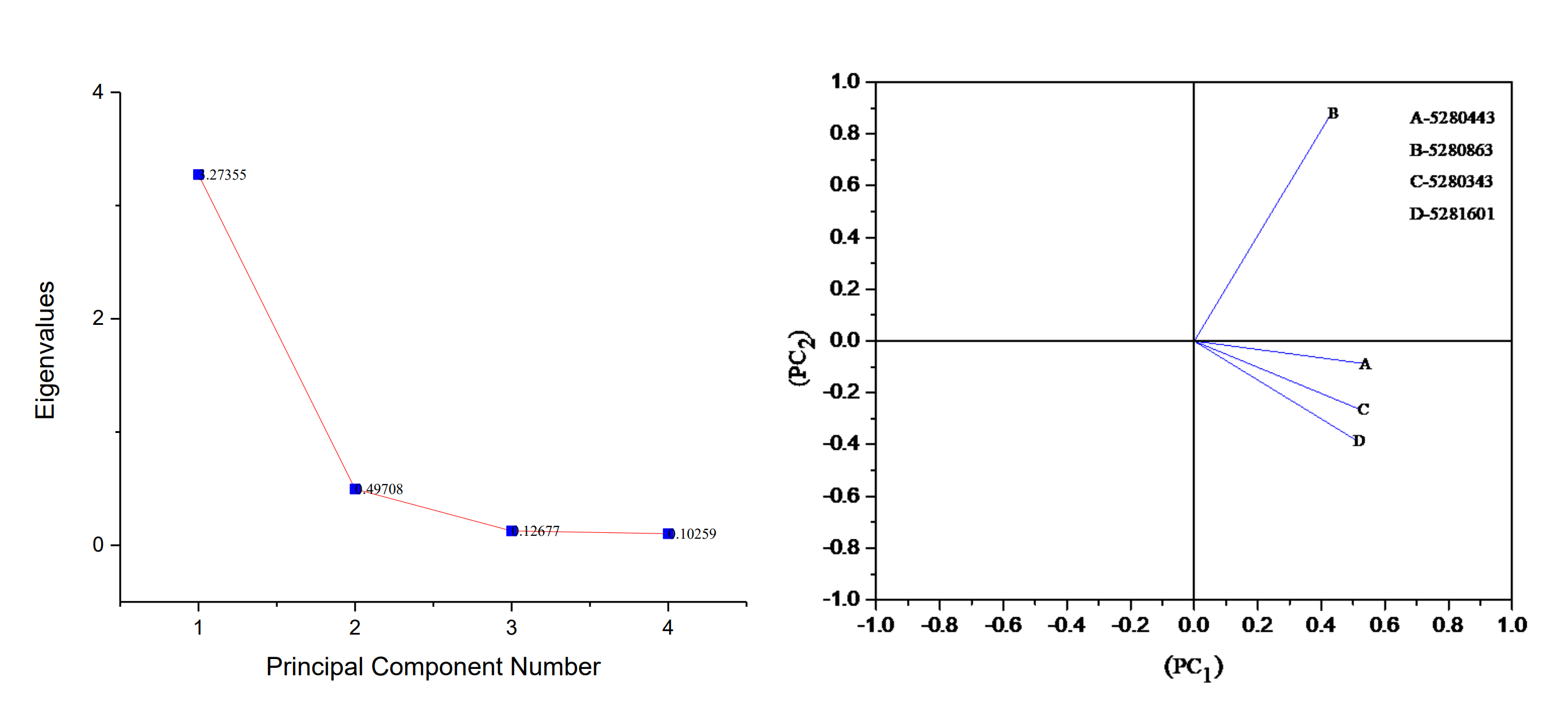


S7. Principal Component Analysis of the native protein and ligands: **5280343 (Quercetin); 5280443 (Apigenin); 5280863 (Kaempferol) and 5281601 (5-hydroxy-7, 4-dimethoxyflavone).**

S1. Chi Vo Van. *Dictionary of Medicinal Plants in Vietnam*. (Medical Publishing House, 1997).

S2. Loi Do Tat. *Vietnamese Medicinal Plants and Remedies*. (Ha Noi Medicine Publishing house, 2004).

S3. Ojo, O. A. *et al.* Deciphering the interactions of compounds from Allium sativum targeted towards identification of novel PTP 1B inhibitors in diabetes treatment: A computational approach. *Informatics in Medicine Unlocked* **26**, 100719 (2021).

S4. Adekiya, T. A., Aruleba, R. T., Klein, A. & Fadaka, A. O. In silico inhibition of SGTP4 as a therapeutic target for the treatment of schistosomiasis. *J Biomol Struct Dyn* **40**, 3697–3705 (2022).
